# Integrin αvβ8-Mediated TGF-β Activation by Effector Regulatory T Cells Is Essential for Suppression of T-Cell-Mediated Inflammation

**DOI:** 10.1016/j.immuni.2015.04.012

**Published:** 2015-05-19

**Authors:** John J. Worthington, Aoife Kelly, Catherine Smedley, David Bauché, Simon Campbell, Julien C. Marie, Mark A. Travis

**Affiliations:** 1 Manchester Collaborative Centre for Inflammation Research, University of Manchester, Manchester M13 9NT, UK; 2 Wellcome Trust Centre for Cell-Matrix Research, Faculty of Life Sciences, University of Manchester, Manchester M13 9PT, UK; 3 Manchester Immunology Group, Faculty of Life Sciences, University of Manchester, Manchester M13 9PT, UK; 4 Immunology Virology and Inflammation Department, CRCL, UMR INSERM1052, CNRS 5286, Centre Léon Bérard, 28 rue Laennec, 69373 Cedex 08 Lyon, France; 5 Université Lyon 1, 69000 Lyon, France; 6 Labex DEVweCAN, 69008 Lyon, France; 7 TGFβ and Immuno-evasion Group, German Cancer Research Center (DKFZ), 69120 Heidelberg, Germany; 8 Gastroenterology Unit, Manchester Royal Infirmary, Central Manchester University Hospital NHS Foundation Trust, Manchester M13 9WL, UK

## Abstract

Regulatory T (Treg) cells play a pivotal role in suppressing self-harmful T cell responses, but how Treg cells mediate suppression to maintain immune homeostasis and limit responses during inflammation is unclear. Here we show that effector Treg cells express high amounts of the integrin αvβ8, which enables them to activate latent transforming growth factor-β (TGF-β). Treg-cell-specific deletion of integrin αvβ8 did not result in a spontaneous inflammatory phenotype, suggesting that this pathway is not important in Treg-cell-mediated maintenance of immune homeostasis. However, Treg cells lacking expression of integrin αvβ8 were unable to suppress pathogenic T cell responses during active inflammation. Thus, our results identify a mechanism by which Treg cells suppress exuberant immune responses, highlighting a key role for effector Treg-cell-mediated activation of latent TGF-β in suppression of self-harmful T cell responses during active inflammation.

## Highlights

• Human and mouse effector Treg cells express functional TGF-β-activating integrin αvβ8
• Treg cell integrin αvβ8-mediated TGF-β activation is not needed for T cell homeostasis
• Integrin αvβ8 expression by Treg cells suppresses active inflammation
• Pathway could be targeted to promote Treg-cell-mediated suppression of inflammation

## Graphical Abstract

**Figure:**
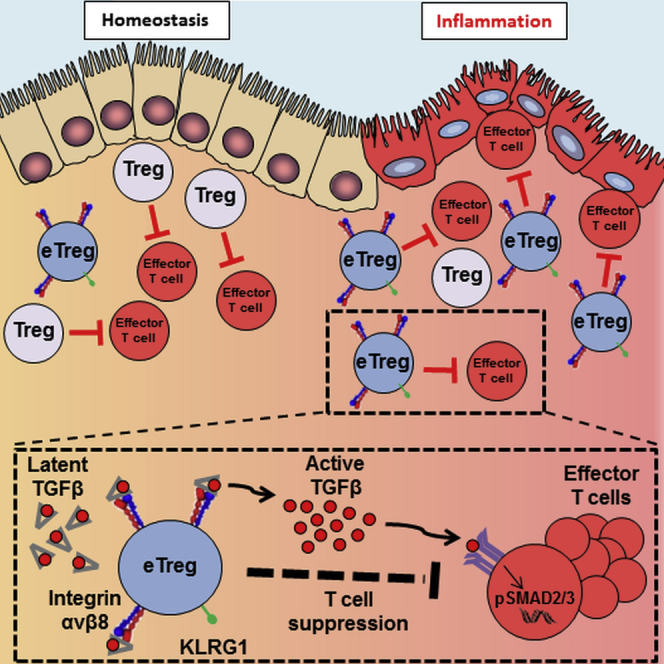

## Teaser

Regulatory T (Treg) cells suppress harmful T cell responses, but mechanisms mediating suppressive function during homeostasis versus inflammation are poorly defined. Travis and colleagues show that activation of the cytokine TGF-β by effector Treg cells, via expression of integrin αvβ8, is crucial for Treg-cell-mediated suppression of inflammatory T cells.

## Introduction

Regulatory T (Treg) cells, a subset of CD4^+^ T cells expressing the transcription factor Foxp3, are crucial in regulating self-harmful T cell responses. Treg cells can develop in the thymus (so-called thymic Treg cells [tTreg cells]) or be induced in the periphery via upregulation of Foxp3 (pTreg cells) (Rudensky, 2011). Mutations in Foxp3 cause severe T-cell-mediated multi-organ inflammation in both mice and humans, highlighting a critical role for Treg cells in maintaining T cell homeostasis. Additionally, the transfer of Treg cells into mouse models of inflammatory disease actively suppresses harmful T cells to prevent inflammation (Maloy and Powrie, 2011). There are current clinical trials using Treg cells that attempt to dampen T cell responses in several human inflammatory disorders (Tang and Bluestone, 2013). Thus, it is paramount that the mechanisms by which Treg cells control immunity be determined, to identify pathways that can be targeted to promote Treg cell function.

A crucial molecule that controls many aspects of Treg cell biology is the cytokine transforming growth factor-β (TGF-β). TGF-β plays a fundamental role in the development of Treg cells, both in the induction pTreg cells (Chen et al., 2003) and in the development of tTreg cells (Konkel et al., 2014; Liu et al., 2008). In addition to roles in Treg cell development, TGF-β plays a fundamental role in the functional ability of Treg cells to suppress T cell responses. Although initial in vitro assays of Treg-cell-mediated suppression both supported and contradicted a functional role for TGF-β (Dieckmann et al., 2001; Jonuleit et al., 2001; Marie et al., 2005; Nakamura et al., 2001; Oida et al., 2006; Takahashi et al., 1998; Thornton and Shevach, 1998), there is now clear evidence that TGF-β plays a key role in mediating Treg cell suppressive function in vivo. Thus, T cells with a reduced capacity to respond to TGF-β cannot be suppressed by Treg cells in mouse models of colitis (Fahlén et al., 2005; Liu et al., 2003) and experimental autoimmune encephalomyelitis (Zhang et al., 2006), and production of TGF-β1 by Treg cells is required for their suppression of colitic T cells in vivo (Nakamura et al., 2004; Pesu et al., 2008). Although some studies have suggested that TGF-β1-deficient Treg cells are still capable of suppressing T-cell-mediated colitis (Fahlén et al., 2005; Kullberg et al., 2005), this suppression is completely abolished by an anti-TGF-β-blocking antibody (Fahlén et al., 2005), again highlighting the crucial role of TGF-β in Treg-cell-mediated suppression in vivo. However, although TGF-β plays a crucial, non-redundant role in suppression of T cells by Treg cells in vivo, how TGF-β is regulated to mediate Treg cell suppressive function is poorly understood.

TGF-β is always produced as an inactive precursor, which must be activated in order to bind its receptor and produce biological function (Worthington et al., 2011a). The *tgfb* gene encodes latency-associated peptide (LAP) upstream of the active TGF-β moiety, which is cleaved from the active cytokine but remains non-covalently attached in a conformation that prevents TGF-β from engaging its receptor. Known activators of TGF-β include a variety of proteases and cell surface molecules that somehow alter the latent complex so that active TGF-β can engage its receptor (Worthington et al., 2011a). Activation of the latent complex is therefore essential for regulation of TGF-β function, yet how TGF-β activation is regulated to control Treg cell suppressive function is completely unknown. Additionally, whether TGF-β plays an important role in Treg-cell-mediated suppression in all situations or whether this pathway is important in only certain immunological settings is a crucial but unanswered question.

Here we show that Foxp3^+^ Treg cells are specialized activators of TGF-β via expression of the integrin αvβ8 and that expression of the integrin is upregulated on activated/effector (e)Treg cells, indicating a potential role in Treg-cell-mediated modulation of active inflammation. Indeed, although lack of expression of integrin αvβ8 on Treg cells does not result in a break in Treg-cell-mediated tolerance at rest, Treg cells lacking expression of the integrin are completely unable to suppress T-cell-mediated inflammation in vivo. These results therefore identify a mechanism by which activated/eTreg cells specifically control inflammatory T cells, highlighting a key role for Treg-cell-mediated activation of latent TGF-β in suppression of self-harmful T cell responses.

## Results

### Treg Cell Expression of the TGF-β-Activating Integrin αvβ8 Is Vital for Suppressing T Cell Expansion

**Figure 1:**
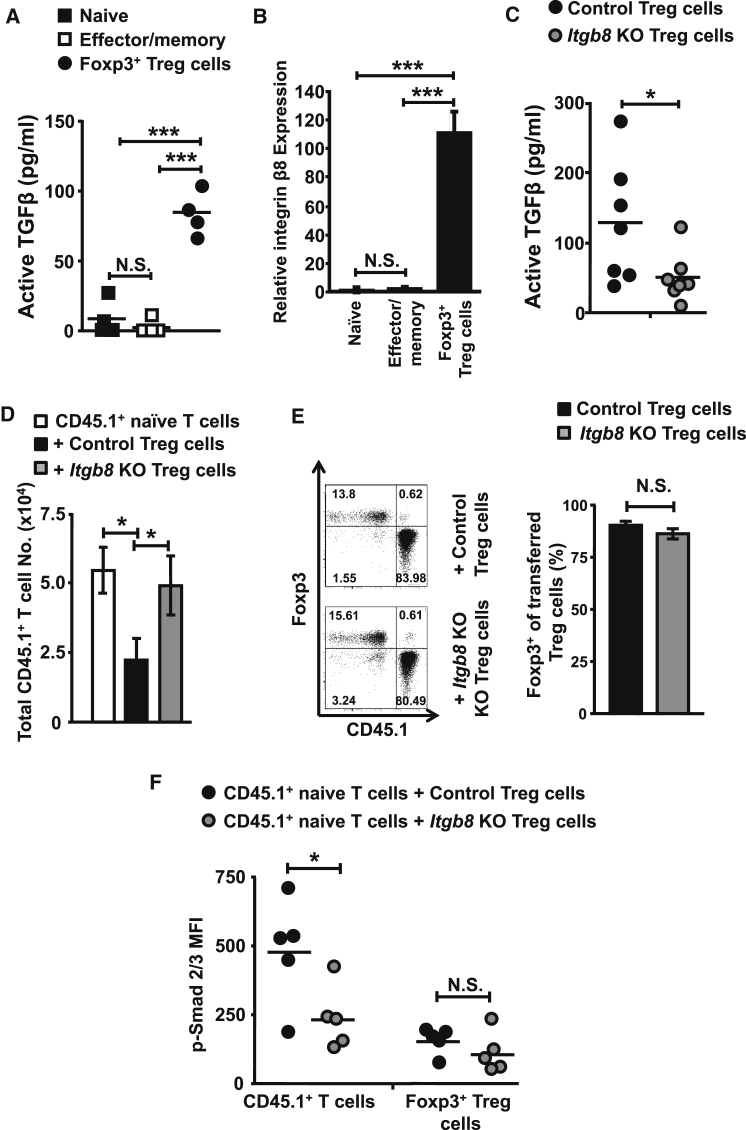
Foxp3^+^ Treg Cells Express the TGF-β-Activating Integrin β8, which Is Essential for Suppression of T Cell Expansion In Vivo (A) TGF-β activation by naive (CD45RB^hi^Foxp3^−^), effector/memory (CD45RB^lo^Foxp3^−^), and Treg (CD45RB^lo^Foxp3^+^) CD4^+^ T cell subsets isolated from the spleen of *foxp3*^GFP^ mice, detected by co-culture with an active TGF-β reporter cell line. Data (n = 4) are from two independent experiments. (B) RNA from naive, effector/memory, and Treg cell CD4^+^ T cell subsets was isolated from the spleen of *foxp3*^GFP^ mice and analyzed for integrin β8 expression by qPCR, with levels normalized to the housekeeping gene *Hprt* and presented relative to naive T cells. Data (n = 2–5) are from five independent experiments. (C) TGF-β activation by control (*Itgb8*^fl/fl^Cre^−^) or *Itgb8* KO (Itgb8^fl/fl^*Cd4*-Cre^+^) splenic Treg cells (CD4^+^CD45RB^lo^CD25^hi^), detected by co-culture with an active TGF-β reporter cell line as in (A). Data (n = 7) are from five independent experiments. (D) Naive (CD4^+^CD45RB^hi^CD25^−^) T cells from CD45.1^+^ congenic mice were transferred into *Rag2*^−/−^ mice at a ratio of 4:1 with control or *Itgb8* KO Treg cells (CD4^+^CD45RB^lo^CD25^hi^). Numbers of transferred CD45.1^+^ naive T cells in the spleen of recipient mice were determined 7 days later. Data (n = 8) are from six independent experiments. (E) Representative flow cytometry plots from (D) and mean percentage Foxp3 expression of transferred Treg cell populations. (F) Analysis of pSmad2/3 expression by flow cytometry in transferred CD4^+^ T cells (CD45.1^+^CD3^+^CD4^+^) and transferred control/*Itgb8* KO Treg cells (CD45.1^−^CD3^+^CD4^+^Foxp3^+^) from spleen 7 days post transfer. Data (n = 5) are from three independent experiments. Error bars represent SEM. See also Figure S1.

Despite the importance of TGF-β in mediating suppression of T cells by Foxp3^+^ Treg cells in vivo, the mechanisms by which this suppression occurs are unknown. Because TGF-β is expressed as a latent complex, we hypothesized that Treg cells could be specialized to activate TGF-β in order to increase local concentrations of the cytokine to mediate suppression. To test this hypothesis, we co-cultured naive CD4^+^ T cells (CD45RB^hi^Foxp3^−^), effector/memory CD4^+^ T cells (CD45RB^lo^Foxp3^−^), and Treg cells (CD45RB^lo^Foxp3^+^) with cells expressing a luciferase reporter for active TGF-β (Abe et al., 1994). Interestingly, Foxp3^+^ Treg cells showed an enhanced ability to activate latent TGF-β compared with both naive and effector/memory T cells (Figure 1A). To determine mechanisms by which Foxp3^+^ Treg cells are specialized to activate TGF-β, we examined Treg cells for molecules that could potentially activate the latent cytokine. We focused on the potential role of integrin αvβ8, which we have previously shown to be expressed by tolerogenic dendritic cells, enabling them to activate TGF-β, a pathway that is crucial in controlling immune homeostasis and responses to infection (Travis et al., 2007; Worthington et al., 2011b, 2013). We analyzed expression of the integrin β8 subunit (which pairs only with integrin αv) at the RNA level, because there are no current antibodies available that recognize murine integrin β8. We found that Foxp3^+^ Treg cells expressed ∼100-fold higher levels of integrin β8 mRNA than naive and effector memory CD4^+^ T cells (Figure 1B). Expression of integrin αv (which pairs with the additional subunits β1, β3, β5, and β6) was similar in all T cell subsets (Figure S1A). Using Treg cells isolated from mice expressing a conditional allele of integrin β8 (*Itgb8*) combined with *Cd4*-Cre (herein called *Itgb8* KO Treg cells) (Travis et al., 2007), we found that such cells no longer displayed the heightened activation of latent TGF-β compared to control Treg cells (Figure 1C). These results suggest that Treg cells activate enhanced levels of TGF-β versus other T cell subsets via expression of integrin αvβ8.

We next tested the suppressive capacity of *Itgb8* KO Treg cells via a model of CD4^+^ T cell expansion in vivo, where naive T cells expand within a few days of transfer into a lymphopenic *Rag2*^−/−^ mouse (Workman et al., 2011). Both control and Treg cells lacking expression of integrin β8 were isolated to similar purities (>94% Foxp3^+^) before transfer (Figures S1B and S1C). We found that *Itgb8* KO Treg cells showed a complete failure to suppress T cell expansion compared to the reduction seen with control Treg cells (Figure 1D). This lack of suppression was not due to any alterations in Treg cell establishment or loss of Foxp3 expression, as shown by the fact that equivalent percentages and Foxp3 expression was found in both transferred control and *Itgb8* KO Treg cells (Figure 1E). In the presence of control Treg cells, we observed a specific increase in phosphorylation of Smad 2/3 (pSmad2/3) in CD4^+^ T cells (Figure 1F), which is the initial signaling event triggered by engagement of TGF-β with its receptor (Worthington et al., 2011a). CD4^+^ T cell pSmad2/3 levels were lower in the presence of *Itgb8* KO Treg cells, indicating that T cells were not seeing as much active TGF-β in the presence of integrin β8-deficient Treg cells (Figure 1F). There was no difference in the amount of pSmad2/3 between the control and *Itgb8* KO Treg cells, indicating that the difference in TGF-β signaling was specific to transferred T cells and not Treg cells at this time point (Figure 1F). Taken together, these data indicate that Foxp3^+^ Treg cells preferentially activate TGF-β via expression of integrin αvβ8 and that this mechanism is essential for the ability of Treg cells to suppress CD4^+^ T cell expansion in vivo.

### Foxp3^+^ Treg Cell Expression of Integrin αvβ8 Is Not Required to Maintain T Cell Homeostasis

**Figure 2:**
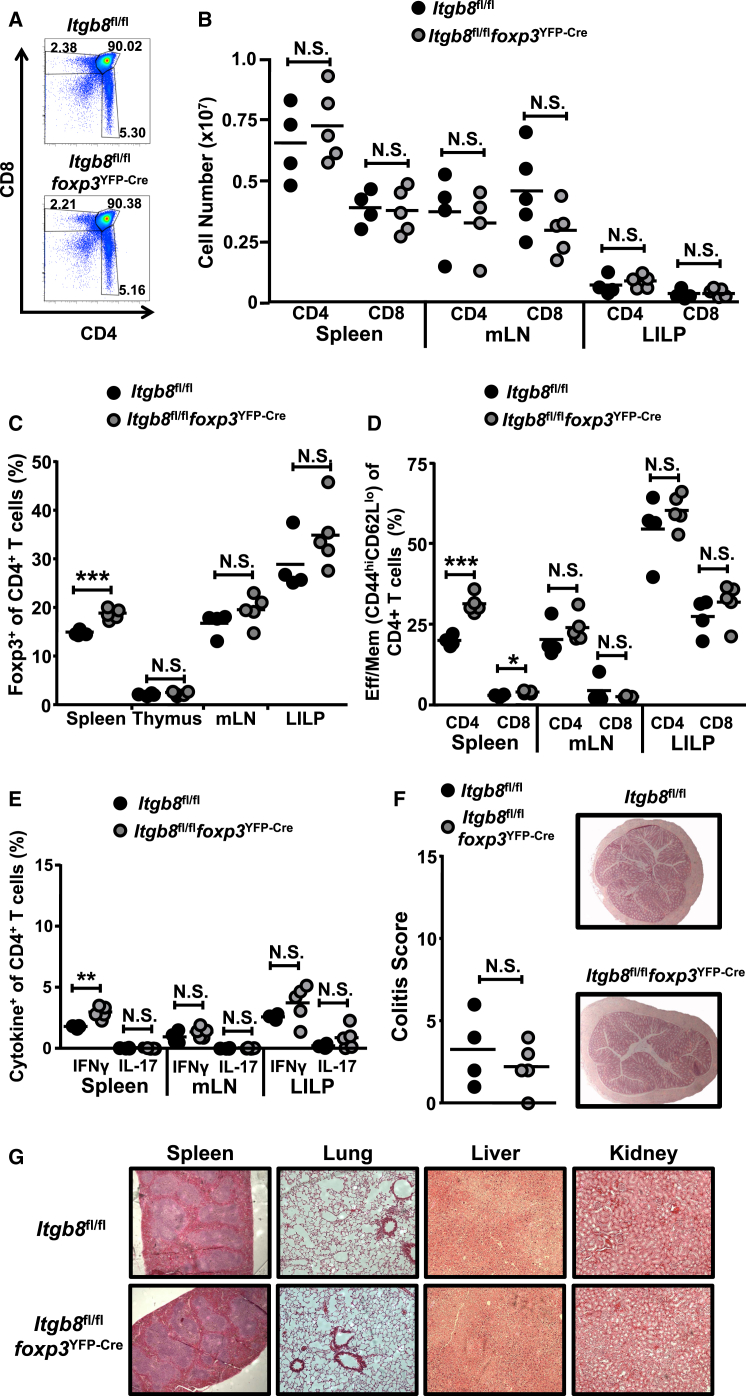
Mice Lacking Foxp3^+^ Treg Cell Expression of Integrin β8 Display Normal T Cell Homeostasis Control mice (*Itgb8*^fl/fl^) and mice lacking expression of integrin β8 on Treg cells (*Itgb8*^fl/fl^*foxp3*^YFP-Cre^), aged 6–12 months old, were examined for potential disruption of T cell homeostasis and inflammation. (A) Representative flow cytometry plots of thymic T cell populations analyzed via CD4/CD8 expression. (B) Spleen, mesenteric LN (mLN), and large intestinal lamina propria (LILP) were examined for T cell cellularity. (C) Spleen, mLN, LILP, and thymus were examined for percent Foxp3^+^ Treg cells. (D and E) Percent CD4^+^ and CD8^+^ effector/memory T cell subsets (CD44^hi^CD62^lo^) (D) and percent intracellular IFN-γ and IL-17 expression by CD4^+^ T cells (E) was examined by flow cytometry. (F) Colitic score and representative images of *Itgb8*^fl/fl^ and *Itgb8*^fl/fl^*foxp3*^YFP-Cre^ mice colon. (G) Representative histological sections of organs from *Itgb8*^fl/fl^ and *Itgb8*^fl/fl^*foxp3*^YFP-Cre^ mice. Data represent n = 4–5.

We have previously demonstrated that mice lacking integrin αvβ8 on all T cells demonstrate no immune pathology at rest (Travis et al., 2007). To directly test the role of this pathway in Treg-cell-mediated control of T cell homeostasis, we crossed mice expressing a conditional allele of integrin β8 with mice expressing *foxp3*^YFP-Cre^ (*Itgb8* flox/flox [fl/fl] × *foxp3*^YFP-Cre^ mice, herein called *Itgb8*^fl/fl^*foxp3*^YFP-Cre^ mice) to specifically delete the integrin on Foxp3^+^ Treg cells (Rubtsov et al., 2008). Despite Treg-cell-expressed integrin αvβ8 being essential for suppression of T cell expansion (Figure 1D), we found that *Itgb8*^fl/fl^*foxp3*^YFP-Cre^ mice developed no spontaneous inflammatory phenotype. Thus, such mice display similar T cell development (Figure 2A) and T cell numbers in spleen and intestinal tissue (Figure 2B) when compared to control mice. There were also similar Treg cell quantities in these compartments, although a slight increase in the splenic Treg cell population was observed (Figure 2C), consistent with a previous report that mice lacking TGF-β signaling specifically on Foxp3^+^ Treg cells show slightly elevated Treg cell numbers (Gutcher et al., 2011). Similar T cell activation and cytokine profiles were observed in intestinal tissue, with a slightly increased T cell activation and CD4^+^ IFN-γ production observed in the splenic T cell population (Figures 2D and 2E). However, this slight increase in splenic T cell activation did not appear biologically significant; mice appeared healthy up until at least 12 months of age and showed no signs of colitis (Figure 2F) or immune pathology in organs examined (Figure 2G). These results indicate that TGF-β activation by Treg-cell-expressed integrin αvβ8 is not required for Treg-cell-mediated control of T cell tolerance at rest.

### Integrin αvβ8 Is Preferentially Expressed on Activated Foxp3^+^ Treg Cells

**Figure 3:**
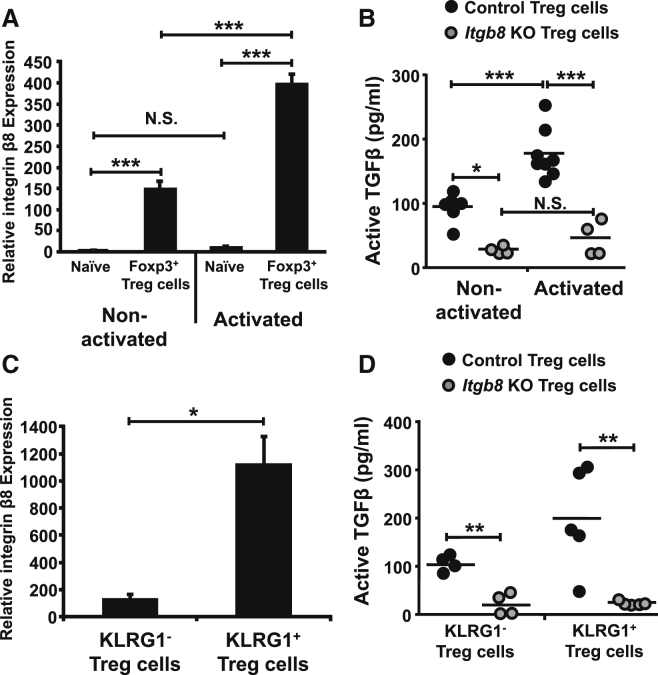
The TGF-β-Activating Integrin αvβ8 Is Preferentially Expressed on Effector Treg Cells (A) RNA from non-activated or anti-CD3 and anti-CD28 antibody-activated naive (CD45RB^hi^Foxp3^−^) or Treg (CD45RB^lo^Foxp3^+^) CD4^+^ T cell subsets isolated from the spleen of *foxp3*^GFP^ mice and analyzed for integrin β8 expression by qPCR. Integrin β8 levels were normalized to the housekeeping gene *Hprt* and presented relative to levels in naive T cells. Data (n = 4–8) are from three independent experiments. (B) TGF-β activation by unstimulated or anti-CD3 and anti-28 antibody-activated splenic control (*Itgb8*^fl/fl^Cre^−^) or *Itgb8* KO (*Itgb8*^fl/fl^*Cd4*-Cre^+^) Treg cells (CD4^+^CD45RB^lo^CD25^hi^), detected by co-culture with an active TGF-β reporter cell line. Data (n = 4–8) are from four independent experiments. (C) Integrin β8 levels were assessed by qPCR via RNA isolated from *KLRG1*^+^ or *KLRG1*^−^ Treg cells (CD45RB^lo^Foxp3^+^, isolated from *foxp3*^GFP^ mice) and values normalized to *Hprt* and displayed relative to naive CD4^+^ T cells in (A). Data (n = 4–8) are from three independent experiments. (D) TGF-β activation levels from *KLRG1*^+^ or *KLRG1*^−^ Treg cell subsets (CD4^+^CD45RB^lo^CD25^hi^KLRG1^+/−^ cells) either control or *Itgb8* KO, assessed by co-culture with a TGF-β reporter cell line as described in (B). Data (n = 4–5) are from three independent experiments. Error bars represent SEM. See also Figure S2.

Given that integrin αvβ8 expression by Treg cells appeared essential for suppression of T cell expansion but redundant for Treg-cell-mediated maintenance of T cell homeostasis, we hypothesized that activation of latent TGF-β by Treg-cell-expressed integrin αvβ8 might play a role during inflammatory responses when both CD4^+^ T cells and Treg cells are activated. We first examined expression of integrin β8 on resting versus activated Treg cell subsets. We found that, whereas activation of naive CD4^+^ T cells did not alter expression of the integrin, activation of Treg cells with anti-CD3 and anti-CD28 antibodies increased expression of integrin β8 (Figure 3A), with integrin αv expression remaining unaltered (Figure S2A). Furthermore, activated Treg cells had increased capacity to activate TGF-β, which was completely dependent on the expression of integrin αvβ8 by activated Treg cells (Figure 3B). We next determined whether integrin αvβ8 was preferentially expressed on activated Treg cell subsets in vivo. Indeed, we found highly elevated expression of integrin β8 mRNA by KLRG1^+^ Treg cells (Figure 3C), which are a subset of Treg cells proposed to represent a terminally differentiated activated/eTreg cell state (Feuerer et al., 2010). The integrin αv was also highly expressed on this KLRG1^+^ eTreg cell population (Figure S2B). Similar to in vitro activated Treg cells, KLRG1^+^ Treg cells showed significantly elevated ability to activate latent TGF-β versus KLRG1^−^ Treg cells, which was completely dependent on the expression of integrin αvβ8 (Figure 3D). Taken together, these data demonstrate that integrin αvβ8 is expressed primarily on activated Treg cells, especially on terminally differentiated eTreg cells.

### Integrin αvβ8 Expression Is Essential for Treg Cell Suppressive Function during Inflammation

Given the elevated expression of integrin αvβ8 on eTreg versus non-eTreg cells, we hypothesized that integrin αvβ8 expression might play an essential role in preventing immunopathology during ongoing inflammation. To address this possibility, we utilized the T cell transfer model of colitis, which acts as an in vivo model for Treg cell suppression of inflammatory T cells (Powrie et al., 1994). In this model, *Rag2*^−/−^ mice receive naive (CD4^+^CD45RB^hi^CD25^−^) T cells, resulting in a wasting disease after 6–8 weeks, which can be reversed by transfer of Treg cells (Maloy et al., 2005).

**Figure 4:**
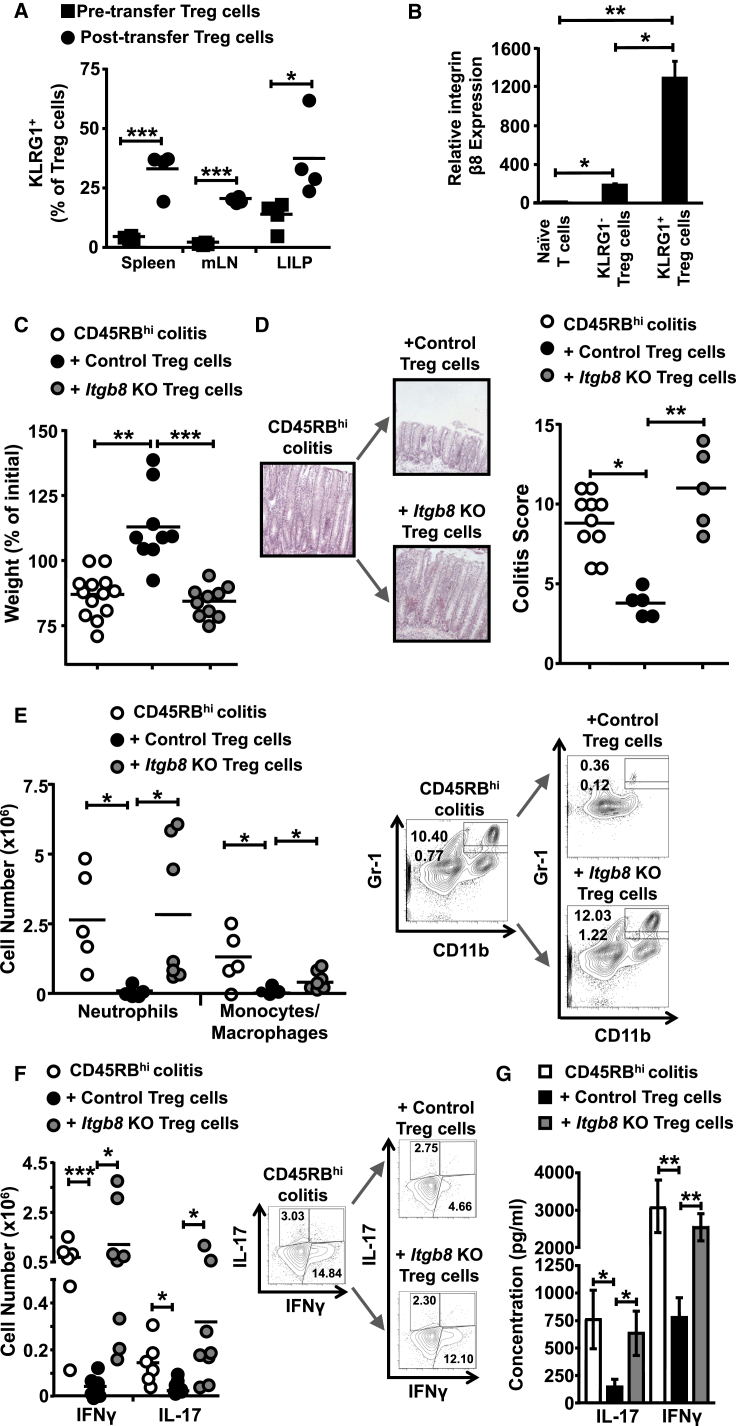
Treg Cells Convert to KLRG1^+^ Effector Treg Cells during Transfer Colitis and Expression of Integrin β8 by Treg Cells Is Essential to Rescue Ongoing Inflammation *Rag2*^−/−^ mice received 0.5 × 10^6^ CD45RB^hi^ T cells alone or followed 2 weeks later by 0.25 × 10^6^ control (*Itgb8*^fl/fl^Cre^−^) or *Itgb8* KO (*Itgb8*^fl/fl^*Cd4*-Cre^+^) Treg cells (CD4^+^CD45RB^lo^CD25^hi^). (A) Expression of KLRG1 on transferred Foxp3^+^ Treg cells (from *foxp3*^GFP^ mice) 6 weeks after transfer versus non-transferred Treg cells. (B) Integrin β8 levels were assessed on *KLRG1*^+^ or *KLRG1*^−^ Treg cells ex vivo by qPCR using RNA isolated from transferred Treg cells (CD4^+^CD45RB^hi^Foxp3^GFP+^) 6 weeks after transfer and values normalized to *Hprt* and displayed relative to naive CD4^+^ T cells. (C and D) Percent of initial mouse weight from time of Treg cell transfer (C) and representative H&E staining and colitic scores of colon samples (D). (E) Neutrophil (Gr1^hi^CD11b^+^) and monocyte/macrophage (Gr1^int^CD11b^+^) populations from the LILP. Total cell number and representative flow cytometry plots are displayed. (F) Intracellular IFN-γ and IL-17 expression in CD4^+^ T cells from LILP. Total cell number and representative flow cytometry plots of data are displayed. (G) IFN-γ and IL-17 cytokine levels from anti-CD3 and anti-CD28 antibody-stimulated total LILP cells, determined via ELISA. Data in (A) and (B) (n = 2–4) are from two independent experiments and in (C)–(G) (n = 5–13) are from four independent experiments. Error bars represent SEM. See also Figure S3.

Given that KLRG1^+^ Treg cells express significantly elevated levels of functional integrin αvβ8 (Figures 3C and 3D), we initially tested whether KLRG1^+^ Treg cell numbers are altered during inflammation. To this end, we examined Treg cell populations during homeostasis compared to Treg cells at 6 weeks after transfer into colitic mice. We found that, compared to Treg cells at rest, the percentage of KLRG1^+^ eTreg cells in the transferred population was significantly increased in splenic, mLN, and intestinal tissues (Figure 4A) and that these KLRG1^+^ Treg cells expressed high levels of integrin β8 mRNA (Figure 4B). Thus, these data indicate that exposure to an inflammatory environment causes an expansion of the KLRG1^+^ eTreg cell subset expressing high amounts of integrin β8.

To directly test the functional role for integrin αvβ8 expression by Treg cells in their ability to suppress inflammatory T cell responses, we tested the suppressive ability of Treg cells lacking expression of integrin β8 in the transfer colitis model. When Treg cells were transferred at a time when inflammation was not yet present (i.e., at the same time as naive T cells), both control and *Itgb8* KO Treg cells completely suppressed weight loss and the development of colitis (Figures S3A–S3D), in agreement with recent findings (Edwards et al., 2014) and the findings that integrin β8 expression on Treg cells does not play a function in preventing inflammation during homeostasis (Figure 2). However, when Treg cells were transferred 2 weeks after naive CD4^+^ T cell transfer into an inflammatory environment, *Itgb8* KO Treg cells completely failed to rescue disease, with weight loss and histological scores being indistinguishable from mice not receiving Treg cells (Figures 4C and 4D). This lack of suppression was in contrast to control Treg cells, which completely suppressed weight loss and inflammation (Figures 4C, 4D, S3E, and S3F).

Previous work has demonstrated that Treg cells suppress both the innate and adaptive arms of the immune response during T-cell-induced colitis (Maloy et al., 2003). However, when transferred 2 weeks after initial naive T cells, *Itgb8* KO Treg cells demonstrated no suppressive ability against either the innate or adaptive immune response, with mice receiving *Itgb8* KO Treg cells showing equivalent numbers of tissue inflammatory monocytes/macrophages (CD11b^+^Gr1^int^), neutrophils (CD11b^+^Gr1^hi^) (Figure 4E; Griseri et al., 2012), and both IFN-γ^+^ and IL-17^+^ CD4^+^ T cells (Figure 4F), as seen in the absence of Treg cells. Indeed, *Itgb8* KO Treg cells failed to reduce the total levels of the inflammatory cytokines IFN-γ and IL-17 produced by cells of the large intestinal lamina propria (Figure 4G).

**Figure 5:**
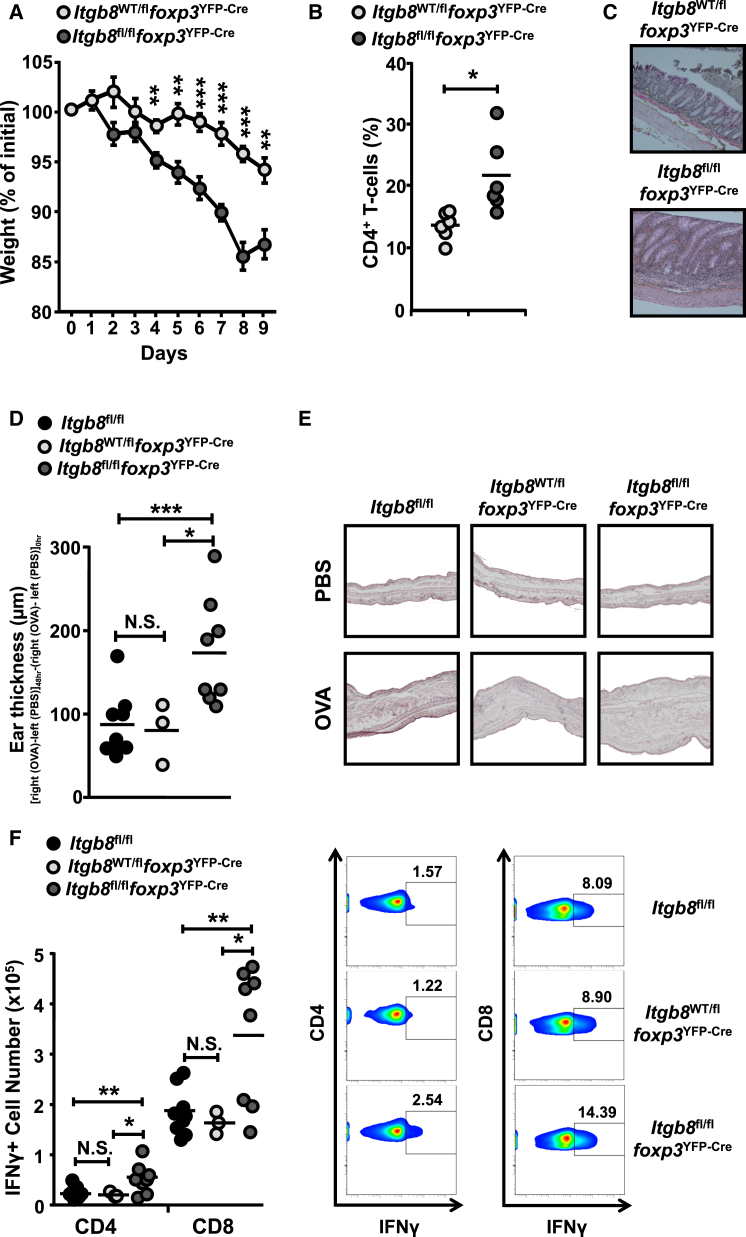
Absence of Integrin β8 on Treg Cells Exacerbates Intestinal and Peripheral Inflammatory T Cell Responses in Lymphocyte-Replete Mice (A–C) Control (*Itgb8*^WT/fl^*foxp3*^YFP-Cre^) and *Itgb8* Treg cell KO (*Itgb8*^fl/fl^*foxp3*^YFP-Cre^) mice received 1% DSS in drinking water for 9 days. (A) Percentage of initial weight from time of treatment. Error bars represent SEM. (B and C) Percentage CD4^+^ T cell populations from the LILP (B) and H&E staining of colon samples (C) at day 9 post-treatment. Data (n = 5) are representative of two independent experiments. (D–F) Control mice (*Itgb8*^fl/fl^ or *Itgb8*^WT/fl^*foxp3*^YFP-Cre^) and mice lacking *Itgb8* expression in Treg cells (*Itgb8*^fl/fl^*foxp3*^YFP-Cre^) were immunized with ovalbumin/CFA followed by subcutaneous challenge with PBS in the left ear pinna and ovalbumin in the right. (D and E) Mean ear thickness (D) and H&E staining of ears (E) 48 hr after challenge. (F) Intracellular IFN-γ expression in CD4^+^ and CD8^+^ T cells from ear draining lymph node. Total cell number and representative flow cytometry plots of data are displayed. Data (n = 6–7) are from two independent experiments.

Next, we determined the function of integrin αvβ8 expression by Treg cells in a non-lymphopenic model of colonic inflammation, via feeding of control (*Itgb8*^WT/fl^*foxp3*^YFP-Cre^) and *Itgb8*^fl/fl^*foxp3*^YFP-Cre^ mice with a low dose of dextran sodium sulfate (DSS). Mice lacking Treg cell integrin β8 expression showed exacerbated colitis compared to control mice expressing Foxp3-YFP-Cre, showing enhanced weight loss (Figure 5A), increased CD4^+^ T cell numbers (Figure 5B), and enhanced pathology (Figure 5C), suggesting an important functional role for integrin αvβ8 expression on Treg cells in controlling intestinal inflammation. Finally, to test whether the suppressive function of integrin αvβ8 expression on Treg cells was specific for suppression of inflammation in the intestine, we induced delayed-type hypersensitivity (DTH) in the ears of mice (Lahl et al., 2007). *Itgb8*^fl/fl^*foxp3*^YFP-Cre^ mice showed exacerbated inflammation compared to controls (both *Itgb8*^fl/fl^ [Cre-negative] and *Itgb8*^WT/fl^*foxp3*^YFP-Cre^ control mice), with enhanced ear thickness (Figure 5D) and inflammation (Figure 5E) and enhanced numbers of CD4^+^ and CD8^+^ T cells producing IFN-γ (Figure 5F). Thus, taken together, these results show that, despite the ability of Treg cells lacking expression of integrin αvβ8 to maintain T cell homeostasis and prevent initial development of inflammation in vivo, expression of this integrin is required for the capacity of Treg cells to suppress effector T cells during inflammation.

### Lack of Integrin αvβ8 Expression Does Not Alter the Homing, Maintenance, or Stability of Treg Cells

**Figure 6:**
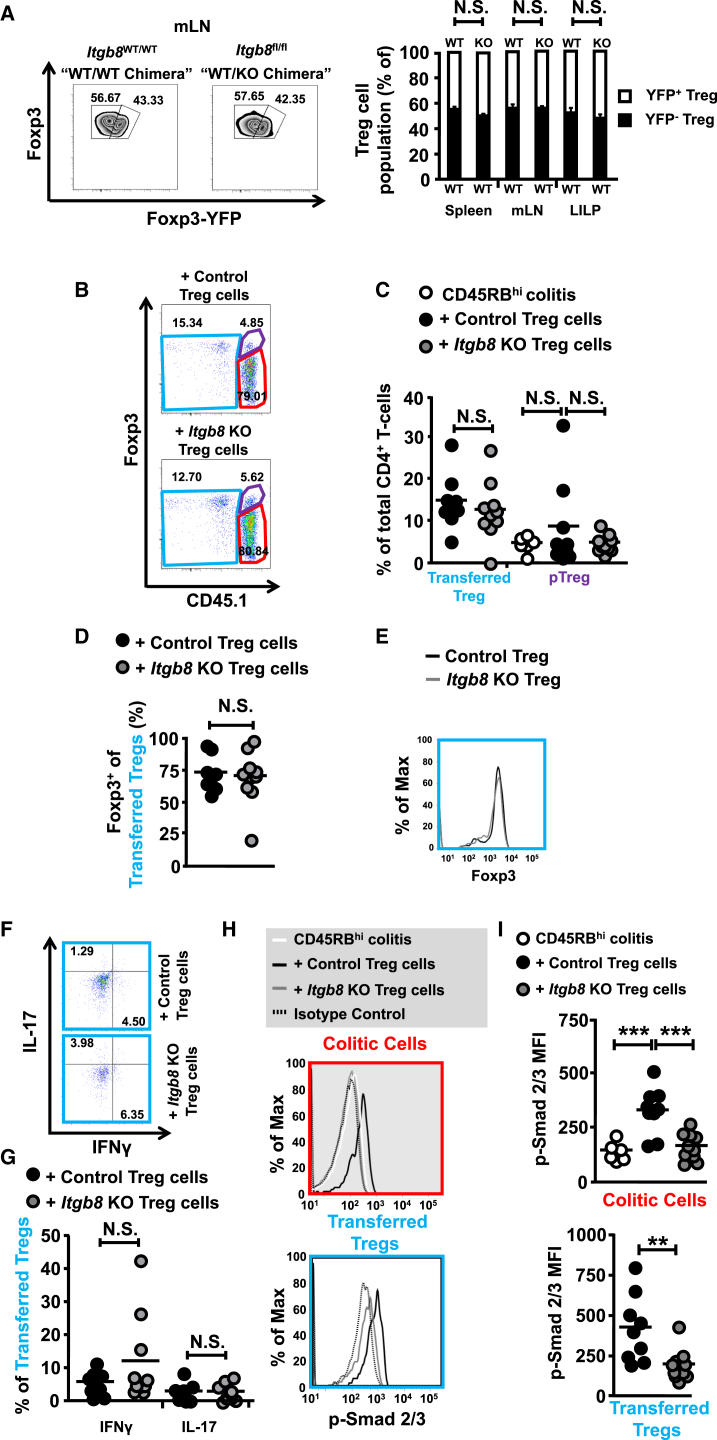
Lack of Integrin β8 Expression by Foxp3^+^ Treg Cells Does Not Alter Treg Cell Maintenance or Stability during Inflammation, but Reduces TGF-β Signaling in Transferred T Cell Populations (A) Analysis of integrin β8-sufficient or KO Treg cell populations in “natural chimera” female mice, heterozygous for *foxp3*^YFP-Cre^ and either *Itgb8*^WT/WT^ or *Itgb8*^fl/fl^. Representative flow cytometry plots of Foxp3^+^YFP-Cre^−^ and Foxp3^+^YFP-Cre^+^ Treg cells from mLN and mean percent population data from spleen, mLN, and LILP. Error bars represent SEM. Data (n = 3–6) are from three independent experiments. (B–I) *Rag2*^−/−^ mice received 0.5 × 10^6^ CD45.1^+^CD45RB^hi^ T cells, followed 2 weeks later by 0.25 × 10^6^ control (*Itgb8*^fl/fl^Cre^−^) or *Itgb8* KO (*Itgb8*^fl/fl^*Cd4*-Cre^+^) Treg cells (CD4^+^CD45.1^−^CD45RB^lo^CD25^hi^). Large intestinal T cell and Treg cell populations were examined 6 weeks later. (B) Representative flow cytometry plots showing Foxp3 expression of transferred naive congenic CD45.1^+^ T cells, CD45.1^−^ control, or *Itgb8* KO Treg cells and gating strategy after analysis of transferred Treg cell (blue), pTreg cell arising from transferred naive T cells (purple), and transferred CD4^+^ T cell (red) populations. (C) Percentage of different Treg cell subsets present (transferred Treg cells and pTreg cells induced from transferred T cells in total T cell population). (D and E) Percentage Foxp3 expression (D) and Foxp3 expression levels (E) in transferred Treg cell populations. (F) Representative flow cytometry plots of intracellular IFN-γ and IL-17 expression in transferred Treg cell populations (blue). (G) Mean percentage expression of IFN-γ and IL-17 in transferred Treg cell population. (H and I) Representative histograms (H) and mean MFI data (I) of pSmad2/3 expression in transferred naive CD45.1^+^CD4^+^ T cells and transferred CD45.1^−^Foxp3^+^ Treg cells. Data (n = 9–10) are from three independent experiments. See also Figure S4 and Tables S1 and S2.

The defect in suppression of active inflammation by *Itgb8* KO Treg cells raised the possibility that lack of integrin αvβ8 expression altered either Treg cell survival, stability, or homing to tissues during homeostasis and/or inflammation. To address this possibility, we first analyzed female mice that were heterozygous for *foxp3*^YFP-Cre^. Because of the location of *foxp3* on the X chromosome and its random inactivation in females, such mice that are homozygous for the integrin β8 floxed allele are a natural chimera for *Itgb8*-expressing and KO Treg cells. We found equivalent ratios of Cre^−^ (Foxp3^+^YFP^−^) and Cre^+^ (Foxp3^+^YFP^+^) Treg cells in all locations tested (spleen, mLN, LILP) between mice that were flox/flox for the *Itgb8* conditional allele (WT/KO chimera) or WT for the *Itgb8* allele (WT/WT chimera) (Figure 6A). Additionally, *Itgb8*-expressing and *Itgb8* KO Treg cells expressed equivalent amounts of a range of Treg-cell-associated markers (Table S1). Together, these data indicate that lack of integrin β8 on Treg cells does not alter the development, maintenance, homing, or stability of Treg cells during homeostasis.

Next, to test whether there was any defect when transferred to an inflammatory environment, we performed transfer colitis experiments, analyzing control or *Itgb8* KO Treg cells transferred to colitic *Rag2*^−/−^ mice that had received naive CD4^+^ T cells 2 weeks earlier. At 6 weeks after transfer, equivalent percentages of both control and *Itgb8* KO Treg cells were observed in the intestine of recipient mice (Figures 6B and 6C), strongly suggesting that integrin αvβ8 expression by Treg cells is not required for either their migration or maintenance in the intestine during inflammation. We also observed equivalent levels of Foxp3^+^ Treg cells arising from the transferred naive T cell population (i.e., induction of pTreg cells) (Figures 6B and 6C), which has previously been shown to rely on TGF-β (Chen et al., 2003), indicating that Treg cell expression of integrin αvβ8 is not involved in the generation of pTreg cells during inflammation. Furthermore, equivalent percentage of Foxp3 expression in control and *Itgb8* KO Treg cells was observed after transfer (Figure 6D), and these cells expressed equivalent quantities of Foxp3 (Figure 6E), indicating that TGF-β activation mediated by integrin αvβ8 on Treg cells is not required for the stability of Foxp3 expression. Indeed, Treg cells lacking expression of integrin αvβ8 did not upregulate expression of the pro-inflammatory cytokines IFN-γ and IL-17 upon transfer (Figures 6F and 6G) and did not cause any pathology when transferred alone to mice (Figures S4A–S4E). Also, despite their failure to rescue disease, *Itgb8* KO Treg cells had a similar capacity to become KLRG1^+^ eTreg cells, with equivalent expression of both KLRG1 and the additional Treg cell activation marker CD103 compared to control Treg cells (Figures S4F–S4G).

Treg cells lacking expression of integrin β8 could not rescue colitis, which meant that control and *Itgb8* KO Treg cells were subjected to different intestinal environments. To determine whether any defects in Treg cell maintenance, stability, or homing were apparent in cells present in the same inflammatory context, we co-transferred a 50:50 mix of β8-expressing (*Itgb8*^WT/WT^*foxp3*^YFP-Cre−^ or *Itgb8*^WT/WT^*foxp3*^YFP-Cre+^) and integrin β8-deficient (*Itgb8*^fl/f^*foxp3*^YFP-Cre+^) Treg cells into colitic mice. 2 weeks later (a time when mice still had intestinal inflammation), the ratio of *Itgb8*-expressing and *Itgb8*-deficient Treg cells was identical to that observed when a 50:50 mix of *Itgb8*^WT/WT^*foxp3*^YFP-Cre−^ and *Itgb8*^WT/WT^*foxp3*^YFP-Cre+^ Treg cells were transferred (Figure S4H). These data strongly indicate that lack of integrin β8 does not affect Treg cell maintenance, stability, or homing in an inflammatory context. Additionally, expression of a range of Treg-cell-associated functional markers showed equivalent expression in both *Itgb8*-expressing and *Itgb8*-deficient Treg cells isolated after co-transfer into colitic mice (Table S2). Finally, when natural chimera mice (female *Itgb8*^fl/fl^ mice heterozygous for *foxp3*^YFP-Cre^) were subjected to either DSS colitis or DTH models, there were no differences in *Itgb8*-expressing (YFP^−^) and *Itgb8*-deficient (YFP^+^) Treg cell ratios compared to equivalent populations in chimeras where all Treg cells expressed integrin β8 (Figures S4I and S4J). Taken together, these data further suggest that lack of functional suppression by Treg cells lacking expression of integrin β8 is not due to defective homing, migration, stability, or activation of these cells.

### Lack of Treg-Cell-Expressed Integrin αvβ8 Dampens TGF-β Signaling in Colitic T Cells and Treg Cells

Because data suggest that TGF-β signalling is required in T cells to permit Treg-cell-mediated suppression in vivo and because activated/eTreg cells activate high levels of TGF-β via integrin αvβ8, we next tested whether colitic T cells showed enhanced TGF-β signalling in the presence of Treg cells in an integrin αvβ8-dependent manner. We found that the transfer of control Treg cells to mice resulted in an increase in pSmad2/3 in transferred CD4^+^ colitic T cells but that this increase in the TGF-β signaling pathway was almost completely absent in the presence of *Itgb8* KO Treg cells (Figures 6H and 6I). Additionally, there was a difference between the transferred control and *Itgb8* KO Treg cells themselves, with reduced levels of pSmad2/3 observed in the *Itgb8* KO Treg cell populations at this time point (Figures 6H and 6I). When T cells and Treg cells were co-transferred together into mice (a situation where *Itgb8* KO Treg cells completely prevented colitis [Figures S3A–S3D]), pSmad2/3 in transferred T cells was equivalent in the presence of both control or *Itgb8* KO Treg cells 6 weeks after transfer (Figure S4K), indicating that during Treg-cell-mediated prevention of colitis, TGF-β signaling in T cells does not require integrin β8 expression on Treg cells. Thus, taken together, our results suggest that expression of integrin αvβ8 is required for Treg-cell-mediated suppression of inflammation via activation of TGF-β, which triggers TGF-β signaling in both colitic CD4^+^ T cells and Foxp3^+^ Treg cells.

### Integrin αvβ8 Is Preferentially Expressed on Human eTreg Cell Populations

**Figure 7:**
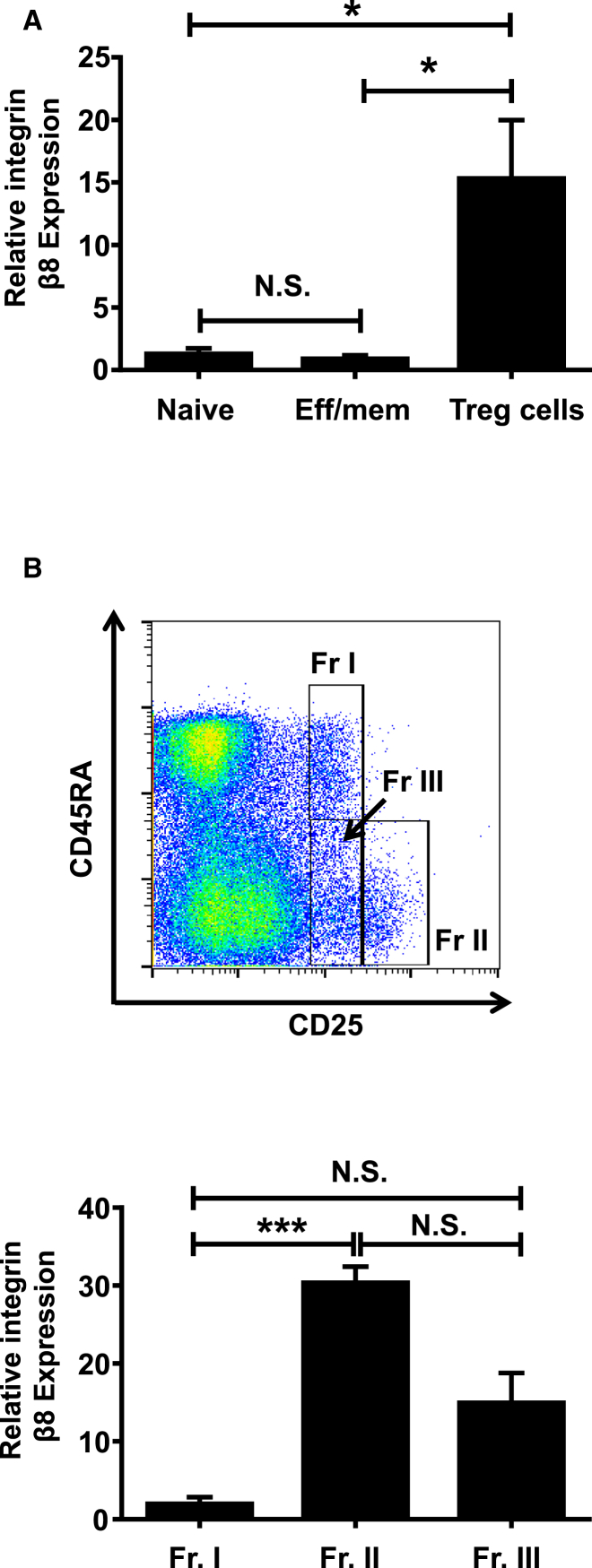
Human Effector Treg Cells Express Integrin β8 (A) RNA was isolated from human peripheral blood naive T cells (CD4^+^CD127^+^CD45RA^+^CD25^−^), effector/memory (CD4^+^CD127^+^CD45RA^−^CD25^−^), and Treg cells (CD4^+^CD127^−^CD25^+^) and integrin β8 expression measured by qPCR. β8 mRNA were normalized to the housekeeping gene *B2M* and presented relative to naive T cell levels (naive, n = 8; effector/memory, n = 7; Treg cell, n = 4). (B) Fr. I CD45RA^+^CD25^++^ (Foxp3^int^) “resting,” Fr. II CD45RA^−^CD25^+++^ (Foxp3^hi^) “activated,” and Fr. III CD45RA^−^CD25^2+^ (Foxp3^int^) Treg cell subsets were sorted and integrin β8 expression measured (Fr. I, n = 3; Fr. II, n = 4; Fr. III, n = 4). Error bars represent SEM. See also Figure S5.

Finally, we addressed whether the expression of integrin αvβ8 seen on mouse Treg cells was mirrored by human Treg cells. We found that, similar to results in mice, Foxp3^+^ Treg cells from human blood preferentially expressed integrin β8 mRNA compared to naive or effector/memory CD4^+^ T cells (Figure 7A). Total human Foxp3^+^ Treg cells also expressed enhanced amounts of the integrin αv subunit (Figure S5A).

We next sought to determine whether expression of integrin αvβ8 was enriched on eTreg cells in humans, similar to our observations in mice (Figure 2). To this end, we isolated Treg cells from the following populations from healthy human blood: the fraction (Fr.) II population (CD45RA^−^ and CD25^hi^) expressing the highest amounts of Foxp3, activation, and suppressive capacity (Miyara et al., 2009), the Fr. I population (CD45RA^+^CD25^int^Foxp3^lo^), which are suppressive and can convert to eTreg cells, and the Fr. III population (CD45RA^−^CD25^int^Foxp3^lo^) which have been reported to produce IL-17 while retaining their suppressive ability (Afzali et al., 2013). All of these Treg cell subsets express the integrin αv subunit (Figure S5B). However, comparable to our data in mice showing highest expression of integrin β8 mRNA on activated/eTreg cells, we found that the human Fr. II effector Treg cell population expresses the highest amounts of integrin β8 (Figure 7B). The intermediately suppressive Fr. III Treg cell population also expresses integrin β8, albeit at lower levels than the more suppressive Fr. II population, whereas the resting Fr. I Treg cell population does not express detectable levels of integrin β8 (Figure 7B). Taken together, these data suggest that, similar to observations in mice, human eTreg cell populations express integrin αvβ8 and this expression directly correlates with their ability to suppress T cell responses (Miyara et al., 2009).

## Discussion

Despite strong evidence indicating that TGF-β plays a non-redundant role in Treg-cell-mediated suppression of T cells in vivo, the mechanisms by which TGF-β is regulated during Treg-cell-mediated suppression are completely unknown. We now demonstrate that Treg cells are specialized to activate latent TGF-β due to expression of the TGF-β-activating integrin αvβ8, with activated/eTreg cells showing highly upregulated expression of the integrin in both mice and humans. Although expression of integrin αvβ8 on Treg cells is not required to maintain T cell tolerance or prevent T-cell-mediated inflammation, it is essential to suppress T cell responses during ongoing inflammation. These results therefore identify a novel mechanism by which activated Treg cells specifically control inflammatory T cells, highlighting a key role for Treg-cell-mediated activation of latent TGF-β in suppression of harmful T cell responses.

Exactly how integrin αvβ8 mediates activation of TGF-β by Treg cells is not clear. Integrin αvβ8 binds to an RGD motif present in LAP, and activation of TGF-β by integrin αvβ8 has been proposed to involve the membrane metalloprotease MMP14 in airway cells (Mu et al., 2002), which cleaves LAP to release active TGF-β. However, we find no evidence for increased expression of MMP14 in either naive Treg cells or eTreg cell subsets compared to naive T cells (data not shown), and therefore the role for MMP14 in promoting enhanced integrin αvβ8-mediated TGF-β activation in Treg cells is unclear. Additionally, whether integrin αvβ8 expression in a T cell is sufficient to convey a regulatory phenotype is unknown, although it is likely that additional factors synergize with the integrin β8-TGF-β pathway to mediate Treg cell suppression during inflammation.

Lack of integrin αvβ8 expression on Treg cells completely abrogated their ability to suppress T cell responses during inflammation, so it was important to determine the mechanism by which Treg cell expression of integrin αvβ8 controlled inflammatory T cells. Treg cells can convert naive T cells into pTreg cells (Andersson et al., 2008; Edwards et al., 2013). Therefore, one possibility for the failure of *Itgb8* KO Treg cells to suppress inflammation was a failure to convert colitic T cells into pTreg cells, thus lacking so-called “infectious tolerance.” Similarly, lack of integrin αvβ8-mediated TGF-β activation could lead to reduced stability of Treg cells, given that TGF-β plays an important role in maintenance of Foxp3 expression (Marie et al., 2005) and the ability of Treg cells that lose Foxp3 expression to become pro-inflammatory “ex-Treg cells” during inflammation (Komatsu et al., 2014; Zhou et al., 2009). However, we observed equivalent percentages of pTreg cells induced in the transferred T cell population in colitis models, and also equivalent percentages of transferred control and *Itgb8* KO Treg cells expressing similar levels of Foxp3, strongly indicating that induction of pTreg cells and stability of transferred Treg cells is not dependent on expression of integrin αvβ8. These findings fit with previous observations in vitro, which suggest that infectious tolerance is independent of the actions of αv integrins (Andersson et al., 2008). Additionally, transfer of *Itgb8* KO Treg cells to *Rag2*^−/−^ mice alone did not result in inflammation, and expression of key additional suppressive markers of Foxp3^+^ Treg cells appeared equivalent between control and *Itgb8* KO Treg cells, indicating that lack of integrin αvβ8 expression by Treg cells does not result in conversion of these cells to a pathogenic phenotype. Instead, we observed that, whereas transfer of control Treg cells into mice receiving naive T cells 2 weeks earlier resulted in induction of TGF-β signaling (phosphorylation of Smad2/3) in colitic T cells, such induction was severely diminished when Treg cells lacked expression of integrin αvβ8. One potential caveat to this observation is that inflammation present when *Itgb8* KO Treg cells do not rescue colitis might affect pSmad2/3 signaling. However, we also show that T cells sense less TGF-β in the presence of *Itgb8* KO versus control Treg cells in settings early after co-transfer of T cells and Treg cells, when no inflammation is present.

We also observed a significant decrease in TGF-β signaling in Treg cells lacking integrin αvβ8, suggesting that Treg cell activation of latent TGF-β can act in an autocrine fashion during inflammation, in addition to acting in a paracrine manner to suppress CD4^+^ T cells. However, Treg cells incapable of responding to TGF-β signaling via the expression of a dominant-negative TGF-β receptor type II (Fahlén et al., 2005), depletion of TGF-β receptor II (Sledzińska et al., 2013), or deficiency in the downstream TGF-β signaling molecule Smad3 (Kullberg et al., 2005) are still able to suppress T-cell-mediated colitis, in contrast to the inability of T cells that cannot sense TGF-β to be suppressed by Treg cells (Fahlén et al., 2005). Thus, taken together with previous studies, our data support a model in which TGF-β, activated by integrin αvβ8 expressed by Treg cells, acts in a paracrine fashion upon CD4^+^ T cells to suppress inflammation.

An important outstanding question is which cells produce the functionally important TGF-β that is activated by Treg-cell-expressed integrin αvβ8? Controversy still exists as to whether TGF-β production by Treg cells is essential for their suppressive function, with different groups showing that Treg cells unable to produce TGF-β either are capable (Fahlén et al., 2005; Kullberg et al., 2005) or incapable (Li et al., 2007; Nakamura et al., 2004) of suppressing T cells in transfer colitis models. Importantly, however, in a model of colitis where it was found that TGF-β1-deficient Treg cells were capable of suppressing inflammation to the same extent as control Treg cells, use of a TGF-β blocking antibody completely abrogated Treg-cell-mediated suppression, showing that TGF-β from non-Treg cell sources is capable of promoting suppression of inflammatory T cells (Fahlén et al., 2005). T cells that cannot express TGF-β1 can still be suppressed by Treg cells in colitis models, suggesting that T-cell-derived TGF-β is redundant for Treg cell function (Li et al., 2007). However, when both T cells and Treg cells cannot make TGF-β, Treg cells show poor suppressive function (Li et al., 2007). Thus, these data suggest that a combination of T-cell- and Treg-cell-produced TGF-β are important in mediating suppression by Treg cells. Given that many different hematopoietic and non-hematopoietic cells produce latent TGF-β and that high levels of latent TGF-β are found throughout the body, we propose that there will be some redundancy in the cell type producing functionally important latent complex for Treg-cell-mediated suppression. However, our data show that integrin αvβ8-mediated activation of latent TGF-β by Treg cells is absolutely required for the Treg-cell-mediated suppression of inflammatory T cell responses.

Another key observation from our study is that, although expression of integrin αvβ8 by Treg cells is essential for Treg-cell-mediated suppression of T cells during ongoing inflammation, this pathway is not essential for Treg-cell-mediated suppression of self-harmful T cells during homeostasis. Mutations in *foxp3* that result in lack of Treg cells result in lethal autoimmune disease in both mice and humans. However, lack of integrin αvβ8 specifically on Treg cells does not result in any overt inflammatory phenotype, strongly indicating that alternative mechanisms contribute to Treg-cell-mediated control of T cell homeostasis. Such mechanisms probably include IL-10-mediated suppression of T cells, given that lack of IL-10 production by Foxp3^+^ Treg cells in mice results in spontaneous inflammation at mucosal surfaces (Rubtsov et al., 2008). Additionally, in agreement with recent work (Edwards et al., 2014), Treg-cell-expressed integrin αvβ8 appears dispensable for inhibition of the initial development of inflammation in models of colitis (when T cells and Treg cells are co-transferred into *Rag2*^−/−^ mice), again suggesting that alternative pathways are important in keeping T cells in check at rest. Interestingly, integrin β8-deficient Treg cells co-transferred with naive T cells into *Rag2*^−/−^ mice still suppress colitis, despite an initial reduction in TGF-β signaling in transferred T cells and an initial reduced ability to control T cell expansion. However, 6 weeks after co-transfer, CD4^+^ T cells show identical heightened pSmad2/3 levels in the presence of integrin β8 KO Treg cells and control Treg cells. Thus, together with data showing that T cells that are refractive to TGF-β signaling cannot be suppressed by Treg cells (Fahlén et al., 2005), these results suggest that, when *Itgb8* KO Treg cells are co-transferred with T cells, there is an alternative source of active TGF-β later after transfer that contributes to TGF-β-mediated suppression of colitic T cells.

We have discovered that integrin αvβ8 expression is upregulated on activated/eTreg cells, specifically on a KLRG1^+^ subset that have been shown to represent a terminally differentiated subset of eTreg cells (Feuerer et al., 2010). This finding fits well with a specific role for Treg-cell-expressed integrin αvβ8 in suppressing T cells during active inflammation, when T cell and Treg cell activation will be prevalent. Indeed, we find that the KLRG1^+^ Treg cell subset is significantly expanded during inflammation. Our work therefore highlights the importance of understanding context-specific pathways by which Treg cells promote suppression in order to better therapeutically target these cells; for example, to promote immune suppression during active disease.

Finally, akin to observations in mice, we find that human Treg cells are specialized to express high levels of integrin αvβ8 and that this expression is enriched on the eTreg cell population. Thus, it appears that the expression profile of integrin αvβ8 translates directly from mouse studies, indicating that this pathway might be a useful therapeutic target in manipulation of human Treg cell suppressive function. Adoptive transfer of human Treg cells is currently in clinical trials for suppression of inflammation in type I diabetes and transplantation (Tang and Bluestone, 2013), so identification of pathways that can be targeted to upregulate the suppressive capacity of these cells are likely to be extremely beneficial.

In conclusion, we have identified that both mouse and human Foxp3^+^ Treg cells express high levels of the integrin αvβ8, which is directly responsible for their ability to activate latent TGF-β, and lack of this pathway results in a failure of Treg cells to suppress T-cell-mediated inflammation. These data not only highlight a novel mechanism by which Treg cells mediate suppression via TGF-β, but also highlight potential specific treatments, via the manipulation of integrin αvβ8, to modulate Treg cell function to promote suppression of inflammation.

## Experimental Procedures

### Animals

Mice lacking T-cell- or Treg-cell-specific expression of integrin β8 were produced via crossing a conditional floxed allele of β8 integrin with *Cd4*-Cre (Travis et al., 2007) or *foxp3*^YFP-Cre^ (Rubtsov et al., 2008; gift from Dr. A. Rudensky, Memorial Sloan-Kettering Cancer Center, New York) mice. *Rag2*^−/−^ and *foxp3*^GFP^ mice were gifts from Dr. K. Okkenhaug (Babraham Institute, Cambridge) and Dr. A. Rudensky, respectively. Mice were maintained in SPF conditions at the University of Manchester and in AniCan, Center Léon Bérard, Lyon, and used at 6 to 12 weeks of age. Stated n numbers are cumulative number of mice used throughout experiments.

### Treg Cell Activation

Treg cells were incubated overnight in 1 μg/ml of anti-CD3 and anti-CD28 antibody, and 5 ng/ml rhIL-2 or as described previously (Edwards et al., 2013).

### TGF-β Activation Assay

T cell subsets were incubated overnight with a TGF-β reporter cell line (Abe et al., 1994) and luciferase activity detected via the Luciferase Assay System (Promega). TGF-β activity was determined as previously described (Worthington et al., 2011b).

### T Cell Transfer Colitis

Naive CD3^+^CD4^+^CD45RB^hi^CD25^−^ T cells were isolated from congenic CD45.1^+^ or WT mice and 0.5 × 10^6^ were injected i.p. into *Rag2*^−/−^ mice alone, with 0.25 × 10^6^ WT or *Itgb8* KO Treg cells (CD3^+^CD4^+^CD45RB^lo^CD25^hi^), or followed 2 or 4 weeks later by 0.25 × 10^6^ WT or *Itgb8* KO Treg cells or a 50/50 mix. Mice were monitored weekly for wasting disease. At 6–8 weeks after initial T cell transfer, 5 × 10^6^ LILP cells were stimulated with anti-CD3 and anti-CD28 antibody (1 μg/ml) and supernatants were examined via ELISA or stimulated overnight with cell stimulation cocktail (eBioscience) and examined for cell populations and intracellular cytokines via flow cytometry.

### Histological Assessment of Inflammation

Tissues were fixed in 4% neutral buffered formalin and lungs were perfused before 5 μm paraffin-embedded sections were cut and stained with hematoxylin and eosin. Colitic inflammation was scored in a blinded fashion using a 0–3 scoring system based on the following five criteria: colon length, crypt hyperplasia and goblet cell depletion, lamina propria leukocyte infiltration, area affected, and severe break down of tissue architecture.

### DSS Colitis Model

Mice received 1% DSS in drinking water and were monitored daily for weight loss before sacrifice at day 9 after treatment, before tissue samples were taken for histology and LILP cells were isolated (see Supplemental Experimental Procedures) and stained via flow cytometry.

### DTH Model

Mice were immunized by s.c. injection of 300 μg Ova (Grade VI, Sigma-Aldrich) in 200 μl PBS/CFA emulsion (Sigma-Aldrich) and challenged 2 weeks later by s.c. injection of 50 μg Ova in 20 μl PBS into the right ear pinna while 20 μl PBS alone was injected into the left. Ear thickness was measured in a blinded fashion prior to and 48 hr after challenge with a caliper micrometer. Ears were split and digested dermis side down in 0.8% Trypsin at 37°C for 30 min before digestion to single-cell suspension in 0.5 Wunch units/ml Liberase TM (Roche) at 37°C for 1 hr. Ear draining lymph nodes were isolated, and single-cell suspensions were re-stimulated and examined via flow cytometry as previously described.

### Statistical Analysis

Results are expressed as mean ± SEM. Where statistics are quoted, two experimental groups were compared via the Student’s t test for non-parametric data. Three or more groups were compared with ANOVA, with Tuckey’s post-test. p < 0.05 was considered statistically significant; ^∗^p < 0.05, ^∗∗^p < 0.01, ^∗∗∗^p < 0.005.

## Author Contributions

J.J.W., A.K., D.B., J.C.M., and M.A.T. designed experiments; J.J.W., A.K., C.S., D.B., and S.C. performed experiments; J.J.W., A.K., S.C., D.B., J.C.M., and M.A.T. interpreted results; and J.J.W., A.K., J.C.M., and M.A.T. wrote the manuscript.

## Supplemental Information

Document S1. Figures S1–S5, Tables S1 and S2, and Supplemental Experimental Procedures

Document S2. Article plus Supplemental Information
