# Supplementary material for: Integrin αvβ8-Mediated TGF-β Activation by Effector Regulatory T Cells Is Essential for Suppression of T-Cell-Mediated Inflammation
Source: Immunity. 2015 May 19;42(5):903–15. doi: 10.1016/j.immuni.2015.04.012 (PMC4448149; doi:10.1016/j.immuni.2015.04.012)
Supplement: Document S1. Figures S1–S5, Tables S1 and S2, and Supplemental Experimental Procedures [file mmc1.pdf]

Immunity
Supplemental Information

Integrin αvβ8-Mediated TGF-β Activation by Effector Regulatory T Cells Is Essential for Suppression of T-Cell-Mediated Inflammation

John J. Worthington, Aoife Kelly, Catherine Smedley, David Bauché, Simon Campbell, Julien C. Marie, and Mark A. Travis

Supplemental Experimental Procedures

Ethics statement

All animal experiments were performed under the regulations of the Home Office Scientific Procedures Act (1986), approved by both the Home Office and the local ethics committee of the University of Manchester and AniCan. The use of human peripheral blood from healthy volunteers was approved by the University of Manchester ethics committee.

Antibodies for flow cytometry

The following antibodies were used: CD4 (clone L3T4; eBioscience), CD45RB (clone C363.16A; eBioscience),CD25 (clone 7D4; Southern Biotech), Foxp3 (clone FJK-16s; eBioscience), IL-17 (clone eBio17B7; eBioscience), IFNγ (clone XMG1.2; eBioscience), CD45.1 (clone A20; Biolegend), CD51 (clone RMV-7; Biolegend), CD8 (clone 53-6.7; eBioscience), IL-10 (clone JES5-16E3; eBioscience), KLRG1 (clone 2F1; KLRG1), CD11c (clone N418; eBioscience), GITR (clone DTA-1; eBioscience), LAP (clone TW7-16B4; eBioscience), GARP (clone YG1C86; eBioscience), ST2 (clone RMST2-2; eBioscience), LAG-3 (clone C9B7W; eBioscience), T-bet (clone 4B10; eBioscience), GATA-3 (clone TWAJ; eBioscience) or p-Smad 2/3 (Santa Cruz). For pSmad2/3 staining, cells were freshly stained and an Alexa Fluor 594-labelled donkey anti-goat secondary antibody was used (Invitrogen).

Flow cytometry staining

Cells were blocked with anti-FcγR antibody (24G2; eBioscience) before labelling with antibodies (See supp. Methods). For intracellular cytokine staining, cells were stimulated overnight with PMA and ionomycin and monensin/Brefeldin A stimulation kit (eBioscience) prior to staining. All samples were analysed on a FACS LSRII flow cytometer.

Quantitative PCR

Total mouse or human RNA was purified from sorted T cell subsets using an RNAeasy Mini/Microkit (Qiagen). Mouse RNA was reverse transcribed using Oligo dT primers, and cDNA for specific genes detected using a SYBR green qPCR kit (Finnzymes). Human RNA was reverse transcribed using GoTaq Reverse Transcription System (Promega) and specific genes detected using TaqMan Universal Master Mix II (with UNG) using the QuantStudio 12K Flex real time PCR system.

Primers for qPCR

Mouse HPRT Forward: GCGTCGTGATTAGCGATGATGAAC,

Mouse HPRT Reverse: GAGCAAGTCTTTCAGTCCTGTCCA,

Mouse Integrin β8 Forward: GGGTGTGGAAACGTGACAAGCAAT,

Mouse Integrin β8 Reverse: TCTGTGGTTCTCACACTGGCAACT.

Human Integrin β8 expression was measured using Taqman gene expression assays (ITGB8 Hs00174456_m1) and normalized to beta 2 microglobulin expression (Hs00984230_m1) (Life Technologies).

Human peripheral blood mononuclear cells (PBMCs) and T cell isolation

Peripheral blood mononuclear cells (PBMCs) were isolated from human leukocyte cones (National Blood Transfusion Service, Manchester, UK) by density centrifugation using Ficoll Paque (VWR International Ltd.). $CD4^+$ T cells were enriched by positive selection using CD4 Microbeads (Miltenyi Biotec) and blocked with normal mouse serum (eBioscience) prior to staining with antibodies against CD45RA (HI100; Biolegend), CD25 (4E3; Miltenyi Biotec) and CD127 (A019D5; Biolegend) for Treg cell subset isolation by flow cytometry cell sorting using the Influx II (BD Bioscience). Foxp3 purity was >95% using anti-Foxp3 antibody (236A/E7, eBioscience).

T cell purification

Splenic T cells were enriched from *foxp3*^{GFP} mice using a Miltenyi $CD4^+$ T cell isolation kit. Enriched cells were antibody labelled before sorting for $CD4^+$, $CD3^+$ naïve ($CD45RB^{hi}Foxp3^-$), effector/memory ($CD45RB^{lo}Foxp3^-$) and Treg cell ($CD45RB^{lo}Foxp3^+$) populations. Alternatively, Treg cells were isolated from control or *Itgb8*$^{fl/f}$*Cd4*-Cre mice via sorting for $CD3^+$ $CD4^+CD45RB^{lo}CD25hi$ cells. Cell purity in all experiments was >99% and $Foxp3^+$ purity from $CD25^{hi}$ isolated Treg cells was confirmed as >95%.

Supplemental Figure Legends

Figure S1 (related to Figure 1). Integrin αv expression on naïve T cells, effector/memory T cells and Foxp3$^+$ Treg cells and gating strategy/purity of Treg cell populations. (A) Representative histograms of integrin αv expression for naïve (CD45RB^{hi}GFP$^-$), effector/memory (CD45RB^{lo}GFP$^-$) and Treg (CD45RB^{lo}GFP$^+$) CD4+ T cells isolated from the spleen of *foxp3^{GFP}* mice, detected by flow cytometry. (B) Representative flow cytometry plots demonstrating sorting purity of obtained naïve (CD45RB^{hi}CD25$^-$) and Treg (CD45RB^{lo}CD25^{hi}) CD4$^+$ T cells. (C) % Foxp3 purity of control (*Itgb8*$^{fl/fl}$Cre$^-$) and *Itgb8* KO Treg cells (*Itgb8*$^{fl/fl}$*Cd4*-Cre$^+$) sorted for transfer experiments. Error bars represent SEM. Data are from eight independent sorts performed.

Figure S2 (related to Figure 3). Integrin αv expression on *in vitro* activated and effector Foxp3$^+$ Treg cells. (A) Representative flow cytometry histograms for integrin αv expression data from control or anti-CD3 and anti-CD28 antibody-activated naive (CD45RB^{hi}GFP$^-$) and Treg (CD45RB^{lo}GFP$^+$) CD4$^+$ T cells isolated from spleens of *foxp3^{GFP}* mice. (B) Representative flow cytometry histograms of integrin αv expression data for splenic KLRG1$^{+/-}$ Treg cells, with pooled data (n = 4) from 2 independent experiments.

Figure S3 (related to Figure 4). Expression of integrin β8 by Treg cells is not required to prevent T cell-induced colitis, and ability of Treg cells to rescue ongoing colitis is not

inhibited by expression of *Cd4*-Cre. (A-D) *Rag2*$^{-/-}$ mice received 0.5x10^{6} CD45RB^{hi} T cells alone or at the same time 0.25x10^{6} control (*Itgb8*$^{fl/fl}$Cre^{-}) or *Itgb8* KO (*Itgb8*$^{fl/fl}$*Cd4*-Cre^{+}) Treg cells (CD4^{+},CD45RB^{lo}, CD25^{hi}). Weight was measured weekly before LILP populations were examined 6 weeks later. (A) Percent of initial mouse weight from time of transfer and (B) representative H&E staining and colitic scores of colon samples. (C) Analysis of neutrophil (Gr1^{hi}CD11b^{+}) and monocyte/macrophage (Gr1^{int}CD11b^{+}) populations from the LILP. Total cell number and representative flow cytometry plots are displayed. (D) Analysis of intracellular IFN-γ and IL-17 expression in CD4^{+} T cells from LILP. Total cell number and representative flow cytometry plots of data are displayed. Data (n=6-8) are from two independent experiments performed. (E-F) *Rag2*$^{-/-}$ mice received 0.5x10^{6} CD45RB^{hi} T cells alone or plus 0.25x10^{6} control Treg cells (from *Itgb8*$^{WT/WT}$*Cd4*-Cre^{+} mice, gated as CD4^{+}CD45RB^{lo}CD25^{hi}) 2 weeks later; weight was measured weekly before LILP populations were examined 6 weeks later. (E) Weights of mice and macroscopic analysis of colon and (F) analysis of cytokine production by CD4+ T cells. Data (n=3).

Figure S4 (related to Figure 6). *Itgb8* KO Treg cells do not directly cause colitis, and lack of integrin β8 expression on Treg cells does not alter their ability to differentiate into effector Treg cells, their numbers during homeostasis and inflammation, or pSmad2/3 signalling when co-transferred with T cells. *Rag2*$^{-/-}$ mice received 0.5x10^{6} control (*Itgb8*$^{fl/fl}$Cre^{-}) or *Itgb8* KO (Itgb8$^{fl/fl}$*Cd4*-Cre^{+}) Treg cells (CD4^{+}CD45RB^{lo}CD25^{hi}); weight was measured weekly before LILP populations were examined 10 weeks later. (A) Percent of initial weight from time of Treg transfer and (B) representative image of large intestine. (C) Analysis of neutrophil (Gr1^{hi}CD11b^{+}) and monocyte/macrophage (Gr1^{int}CD11b^{+}) populations. Total cell

number and representative flow cytometry plots are displayed. (D) Percentage mean of Foxp3 expression in transferred T cell populations. Error bars represent SEM. (E) Analysis of intracellular IFN-γ and IL-17 expression in transferred Treg cells. Total cell number and representative flow cytometry plots of data are displayed. Data in A-E (n=3-5) are from two independent experiments performed. (F-G) *Rag2*$^{-/-}$ mice received 0.5x10^{6} CD45.1^{+} CD45RB^{hi} T cells, followed 2 weeks later by 0.25x10^{6} control or *Itgb8* KO Treg cells (CD4^{+}CD45.1^{+}CD45RB^{lo}CD25^{hi}). Large intestinal T cell and Treg cell populations were examined 6 weeks later. (F) Representative flow cytometry plots of transferred control and *Itgb8* KO Treg cell populations analysing effector Treg cell (CD103^{+}, KLRG1^{+}) markers. (G) Percentage KLRG1 expression levels in transferred Treg cell populations in spleen, mLN and LILP. Data (n=9-10) are from three independent experiments performed. (H) *Rag2*$^{-/-}$ mice received 0.5x10^{6} CD45RB^{hi} T cells, followed 4 weeks later by transfer of 0.25x10^{6} Treg cells, consisting of a 50/50 mix of WT Cre-negative (*Itgb8*$^{WT/WT}$*foxp3*$^{YFP\text{-}Cre-}$) and *Itgb8* KO Treg cells (*Itgb8*$^{fl/fl}$*foxp3*$^{YFP\text{-}Cre+}$) or WT (*Itgb8*$^{WT/WT}$*foxp3*$^{YFP\text{-}Cre-}$) and WT (*Itgb8*$^{WT/WT}$*foxp3*$^{YFP\text{-}Cre+}$) Treg cells (CD4^{+}CD45RB^{lo}CD25^{hi}). Treg cell populations from spleen, mLN and LILP were examined 2 weeks later for the ratio of YFP^{-}/YFP^{+} Foxp3^{+} transferred Treg cells via flow cytometry. Data (n=3-6) from 3 independent experiments. Error bars represent SEM. (I-J) Analysis of Foxp3^{+} Treg cells, YFP^{-}/YFP^{+} ratio in female mice heterozygous for *foxp3*$^{YFP\text{-}Cre}$ and either *Itgb8*$^{WT/WT}$ or *Itgb8*$^{fl/fl}$ during inflammation induced by (I) 1% DSS or (J) DTH. Treg cell populations from mLN and LILP (I) or ear-draining LN (aLN) and ear (J) were analysed via flow cytometry. Red lines on graphs indicate mean ratio at homeostasis. Error bars represent SEM. (Data n=3-5). (K) *Rag*2$^{-/-}$ mice received 0.5x10^{6} CD4^{+}CD45.1^{+}CD45RB^{hi} T cells alone or at the same time as 0.25x10^{6} control (*Itgb8*$^{fl/fl}$Cre^{-}) or *Itgb8* KO (*Itgb8*$^{fl/f}$*Cd4*-Cre^{+}) Treg cells (CD4^{+}CD45RB^{lo}CD25^{hi}). Mean

MFI data of pSmad2/3 expression in transferred naive CD45.1^{+}CD4^{+} T cells and transferred CD45.1^{-} Foxp3^{+} Treg cells. Data (n=4-8) are from two independent experiments performed.

Figure S5 (related to Figure 7). Human T cell expression of integrin αv. (A) RNA was isolated from human peripheral blood naïve T cells (CD4^{+}CD127^{+}CD45RA^{+}CD25^{-}), effector/memory (CD4^{+}CD127^{+}CD45RA^{-}CD25^{-}), and Treg cells (CD4^{+}CD127^{-}CD25^{+}) and integrin αv expression measured by qPCR. Integrin αv levels were normalized to the housekeeping gene *B2M* and presented relative to naïve T cell levels (naïve, n=6, effector/memory n=6; Treg cells n=4). Error bars represent SEM. (B) Fr. I CD45RA+CD25^{++} (Foxp3^{int}) "resting", Fr. II CD45RA^{-}CD25^{+++} (Foxp3^{hi}) "activated" and Fr. III CD45RA^{-}CD25^{++} (Foxp3^{int}) Treg cell subsets were sorted and integrin αV expression measured (Fr. I n=3; Fr.II n=3; Fr.III n=2). Error bars represent SEM.

Table S1 (related to Figure 6). Expression of Treg cell-associated markers on control and *Itgb*8 KO Treg cells in "natural chimera" female mice. Flow cytometric analysis of control (YFP-Cre^{-}) and *Itgb8* KO (YFP-Cre^{+}) Treg cells isolated from spleen, mLN and LILP of female 'natural chimera' mice heterozygous for *foxp3*$^{YFP\text{-}Cre}$ and homozygous for the *Itgb8* flox allele. As a control for *foxp3*$^{YFP\text{-}Cre}$ expression, YFP-Cre^{+} Treg cells from *Itgb8*$^{WT/WT}$*foxp3*$^{YFP\text{-}Cre}$ female 'natural chimera' mice, heterozygous for *foxp3*$^{YFP\text{-}Cre}$ (control (YFP-Cre^{+})) were also analysed. Data n=3-6 from 3 independent experiments. % means± (SEM) (to 2 d.p.) or mean fluorescent intensity, indicated in italics, ± (SEM) (to nearest whole number).

Table S2 (related to Figure 6). Lack of integrin β8 expression by Foxp3+ Treg cells does not alter expression of classical Treg cell effector molecules during inflammation. *Rag2*$^{-/-}$ mice received 0.5x10^{6} CD45RB^{hi} T cells, followed 4 weeks later by transfer of 0.25x10^{6} Treg cells, consisting of a 50/50 mix of WT (*Itgb8*$^{WT/WT}$Cre^{-}) and *Itgb8* KO Treg cells (*Itgb8*$^{fl/fl}$*foxp3*$^{YFP-Cre+}$) or a mix of WT Cre^{-} (*Itgb8*$^{WT/WT}$*foxp3*$^{YFP-Cre-}$) and WT Cre^{+} (*Itgb8*$^{WT/WT}$*foxp3*$^{YFP-Cre+}$) Treg cells (CD4^{+}CD45RB^{lo}CD25^{hi}). Treg cell populations from spleen, mLN and LILP were examined 2 weeks later. Flow cytometric analysis was performed for control (YFP-Cre^{-}) and *Itgb8* KO (YFP-Cre^{+}) Treg cells and also for *Itgb8*-expressing Treg cells expressing *foxp3*$^{YFP-Cre}$ as a control for Cre expression (control (YFP-Cre^{+})). Data n=3-6 from 3 independent experiments, % means± (SEM) (to 2 d.p.) or mean fluorescent intensity, indicated in italics, ± (SEM) (to nearest whole number).

FIGURE S1 (related to Figure 1)

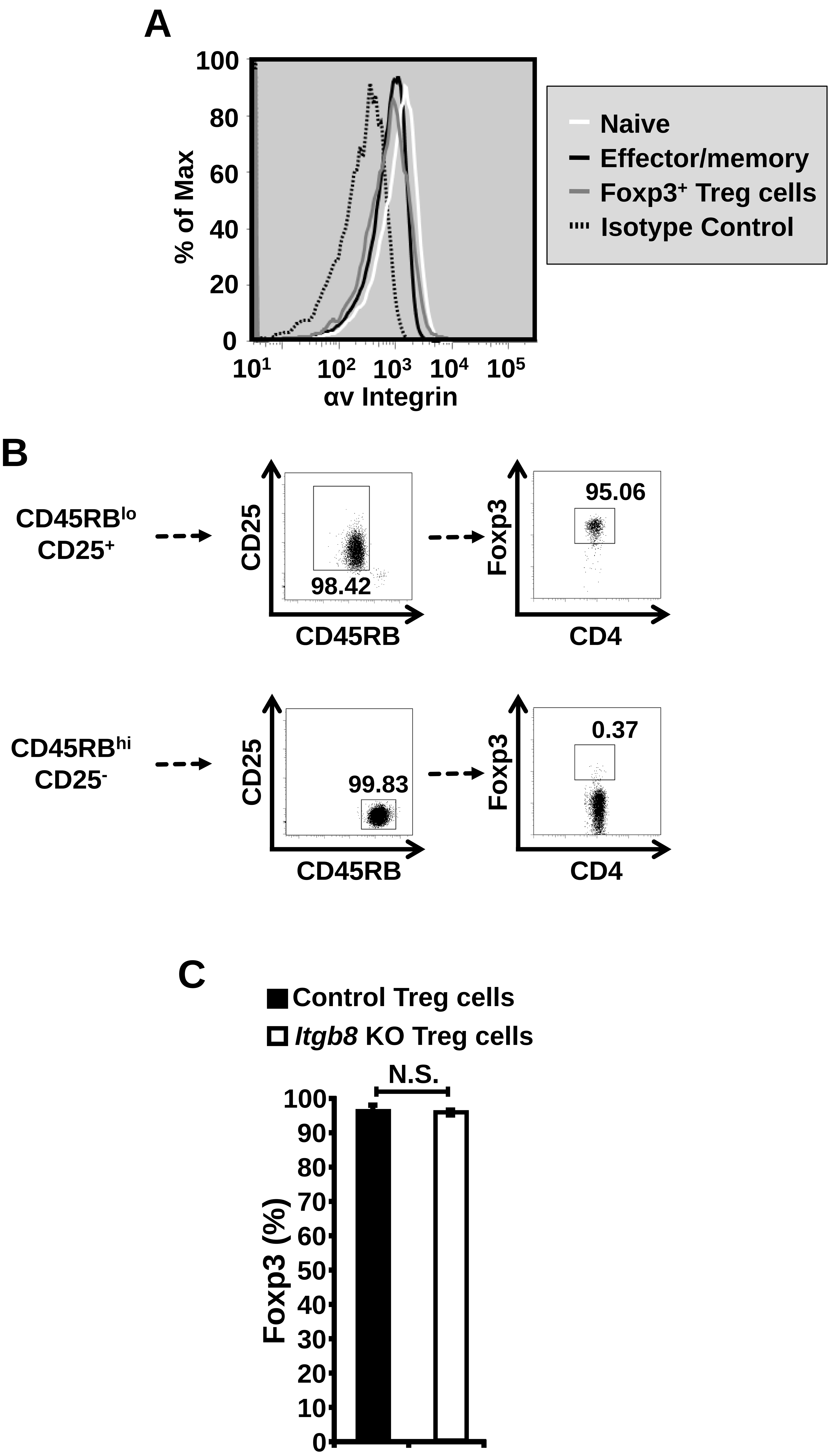

FIGURE S2 (related to Figure 3)

A

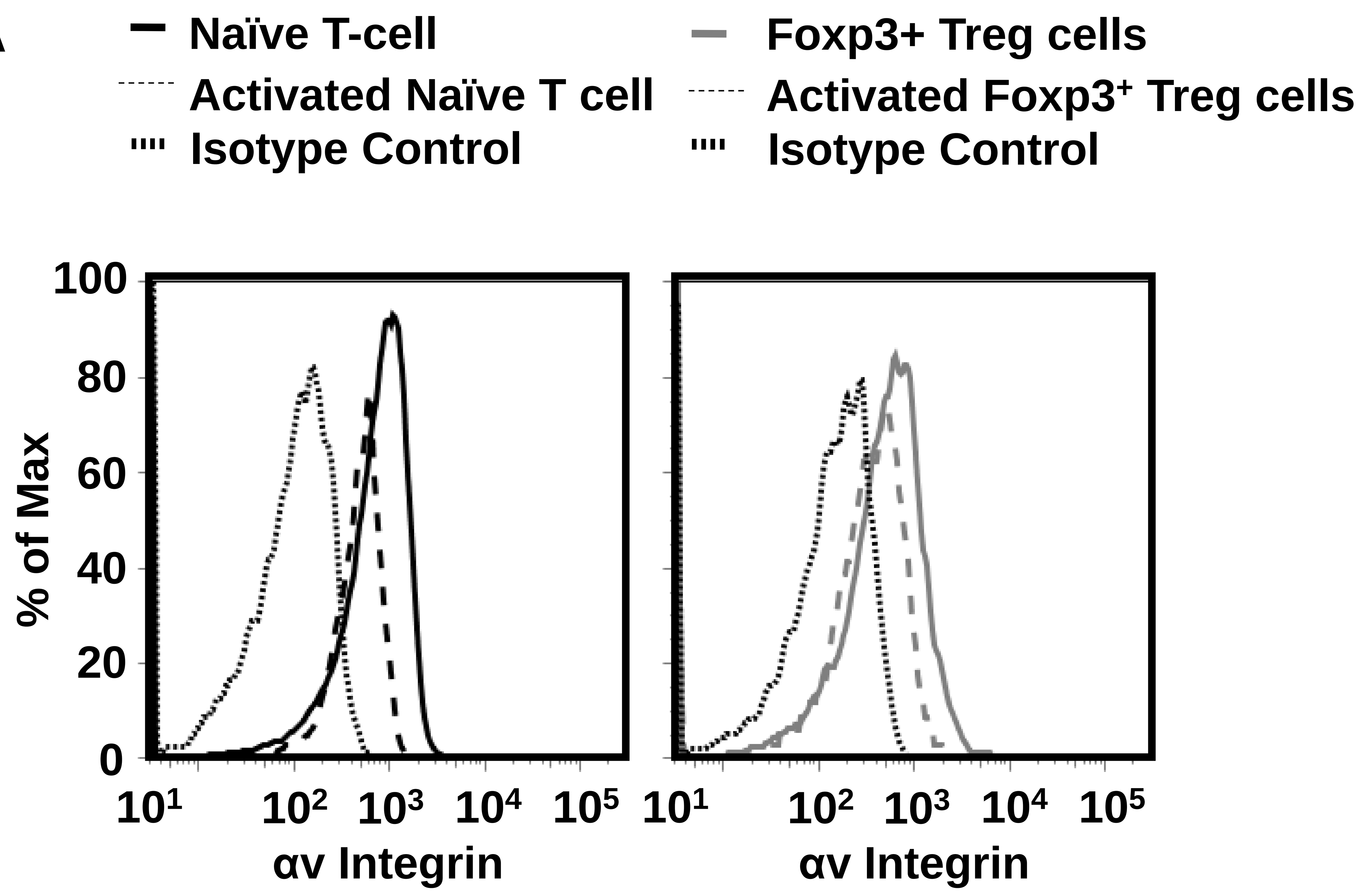

B

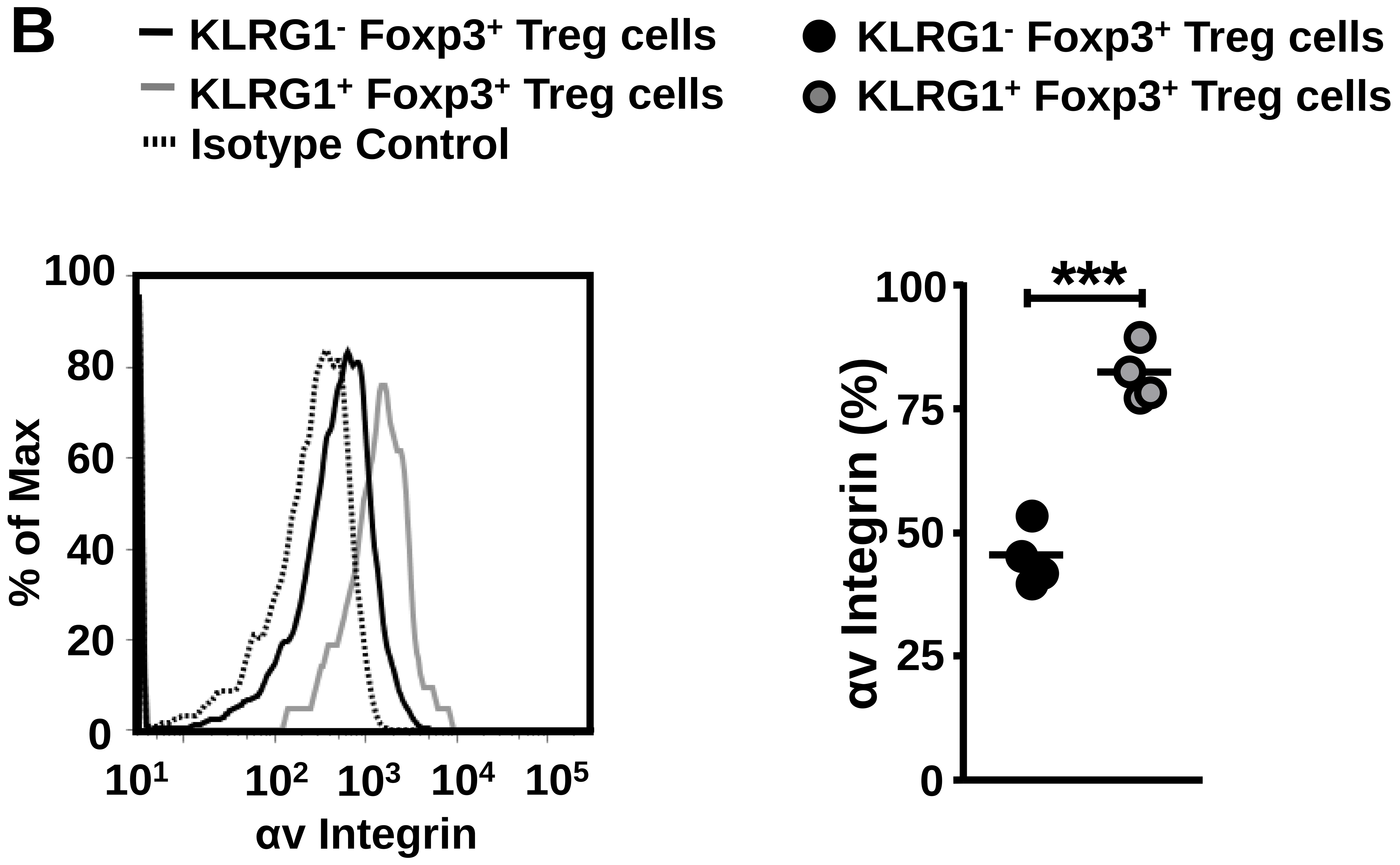

FIGURE S3 (related to Figure 4)

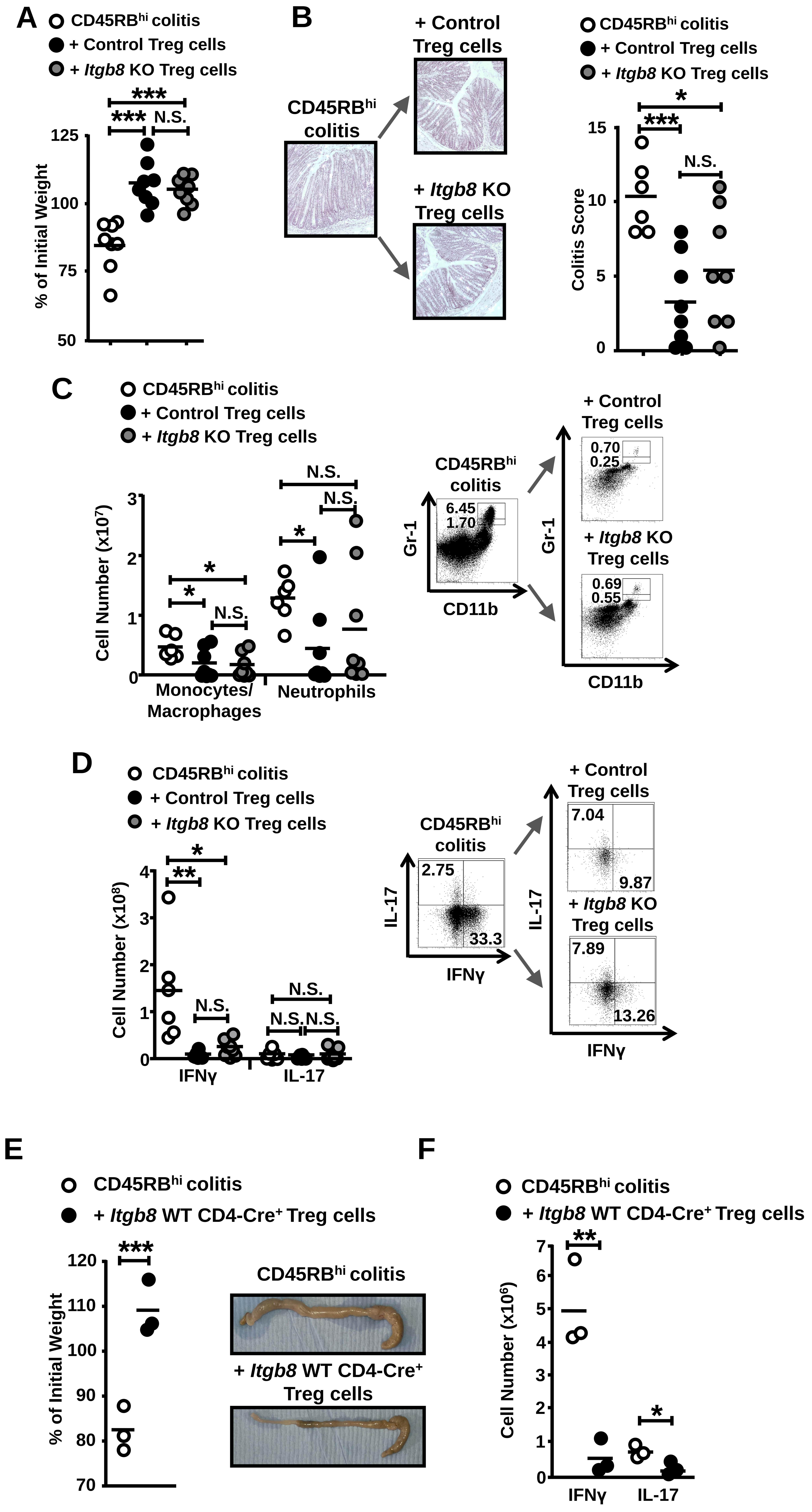

FIGURE S4 (related to Figure 6)

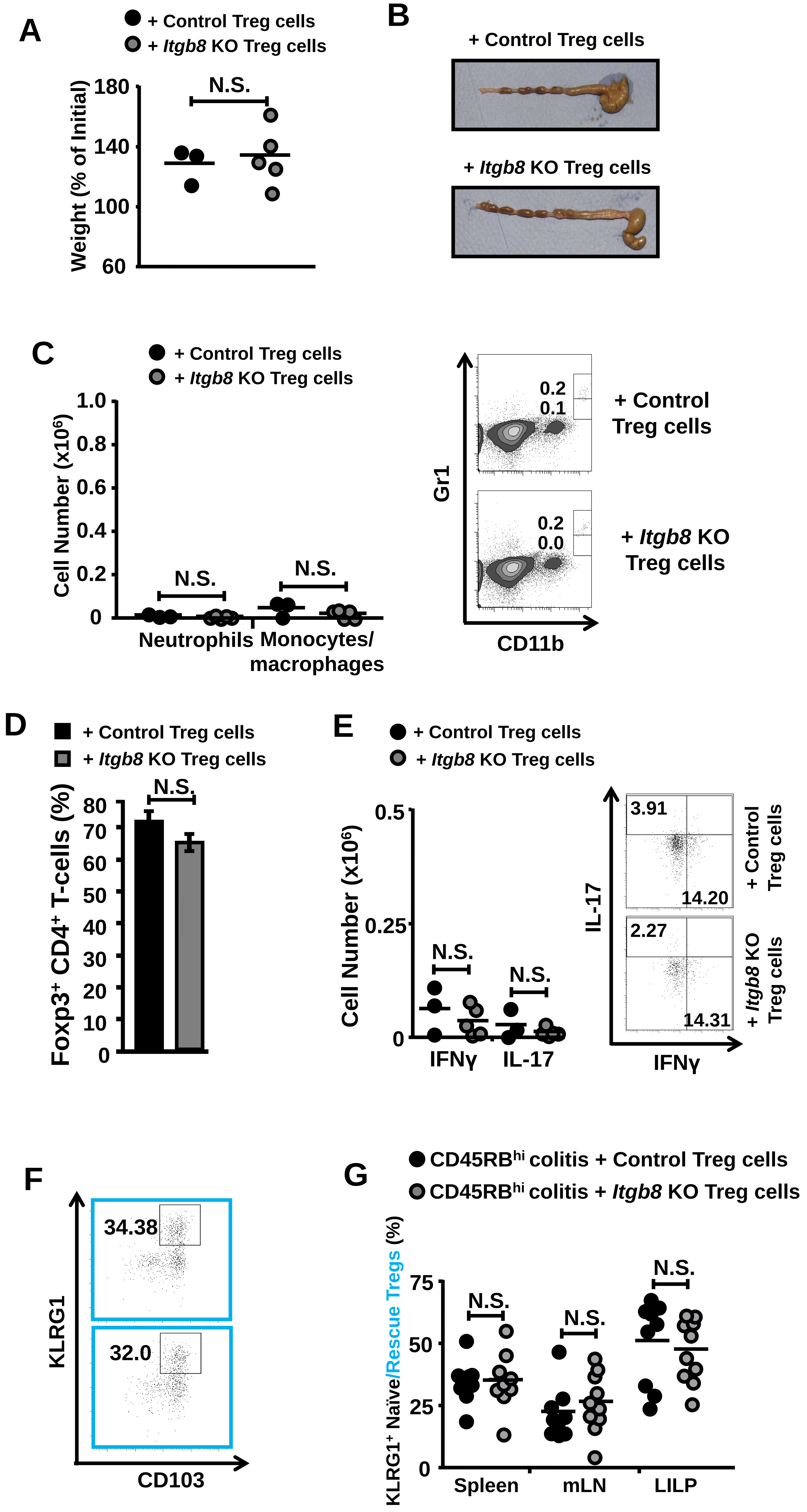

FIGURE S4 continued (related to Figure 6)

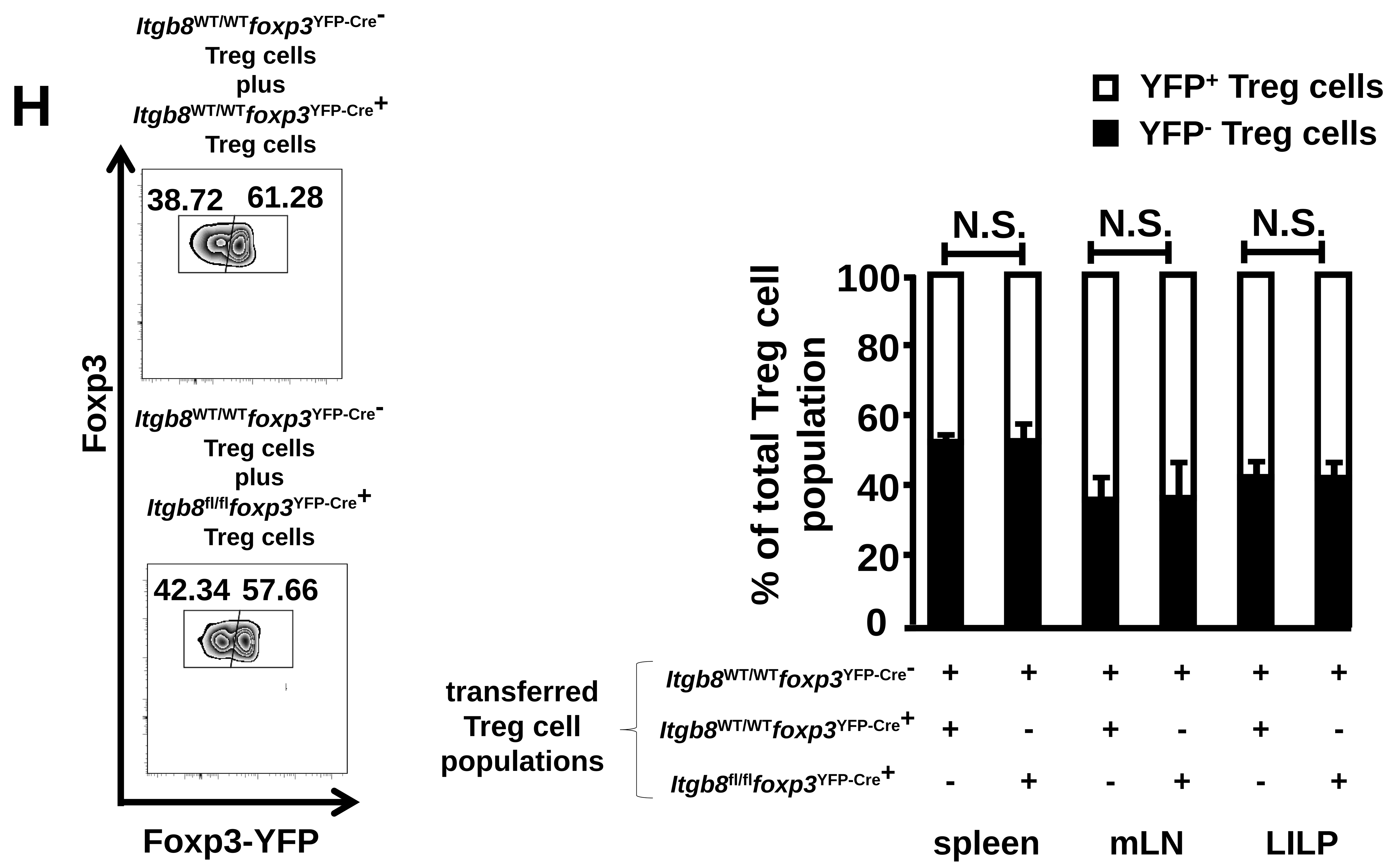

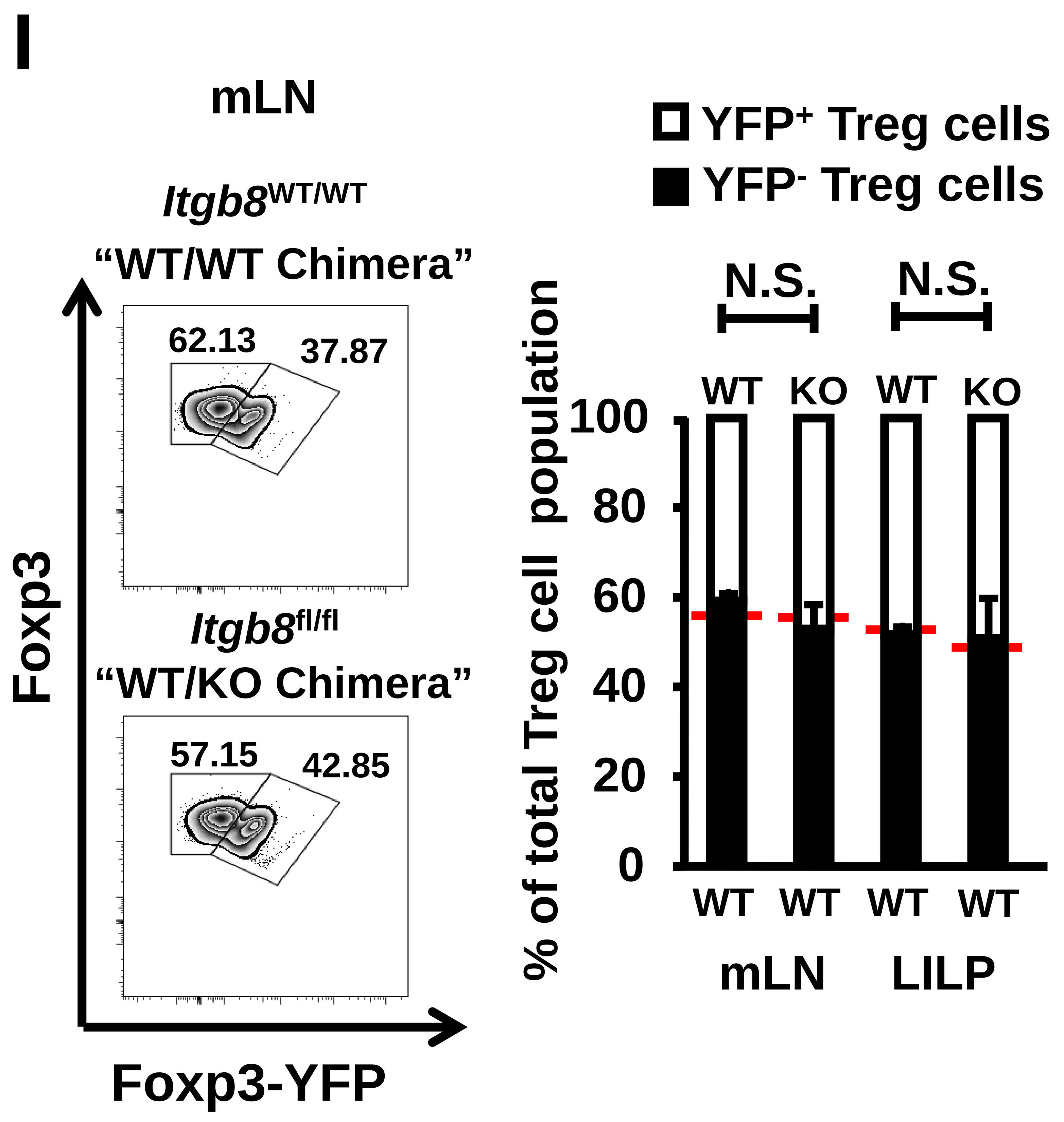

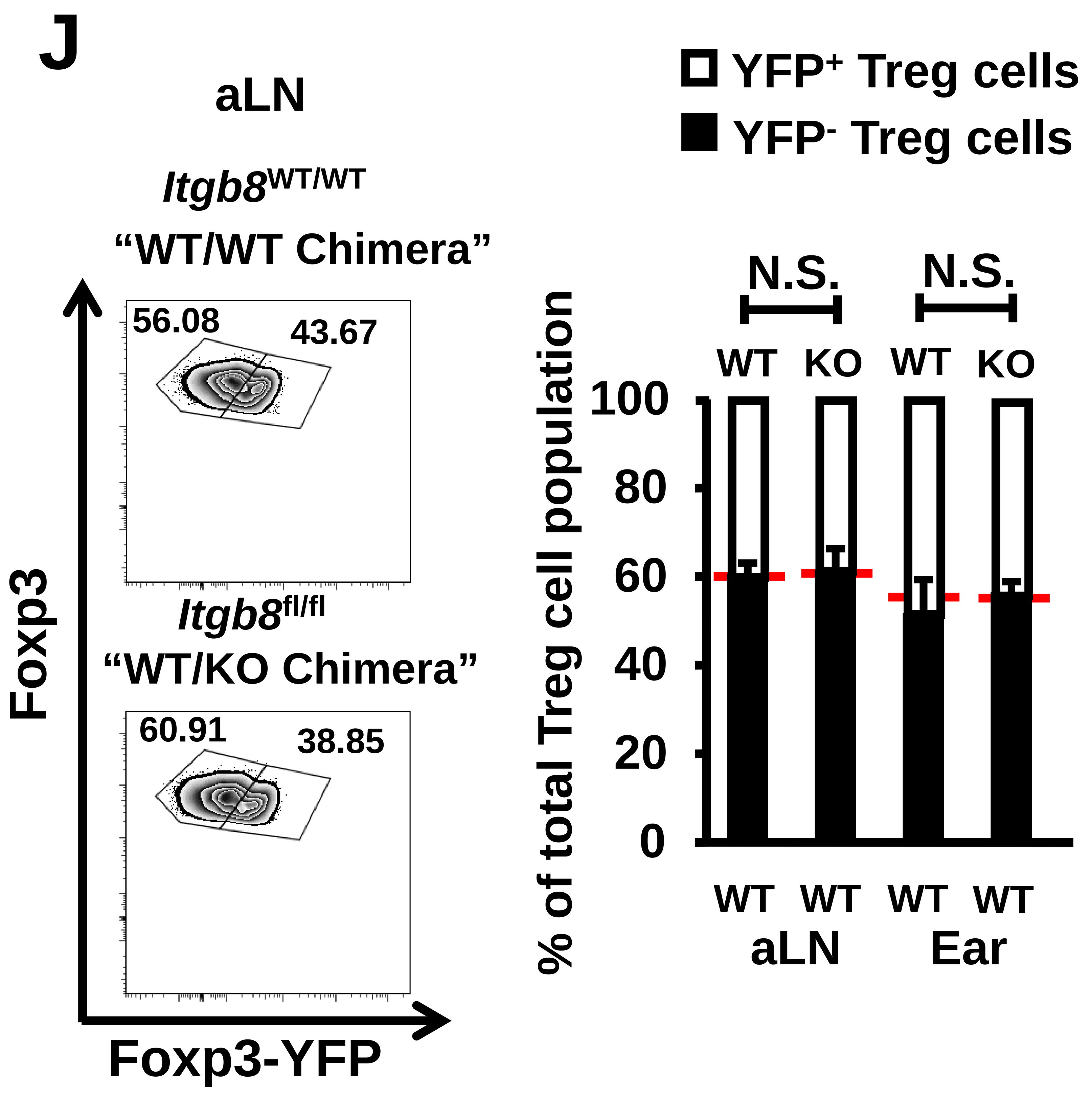

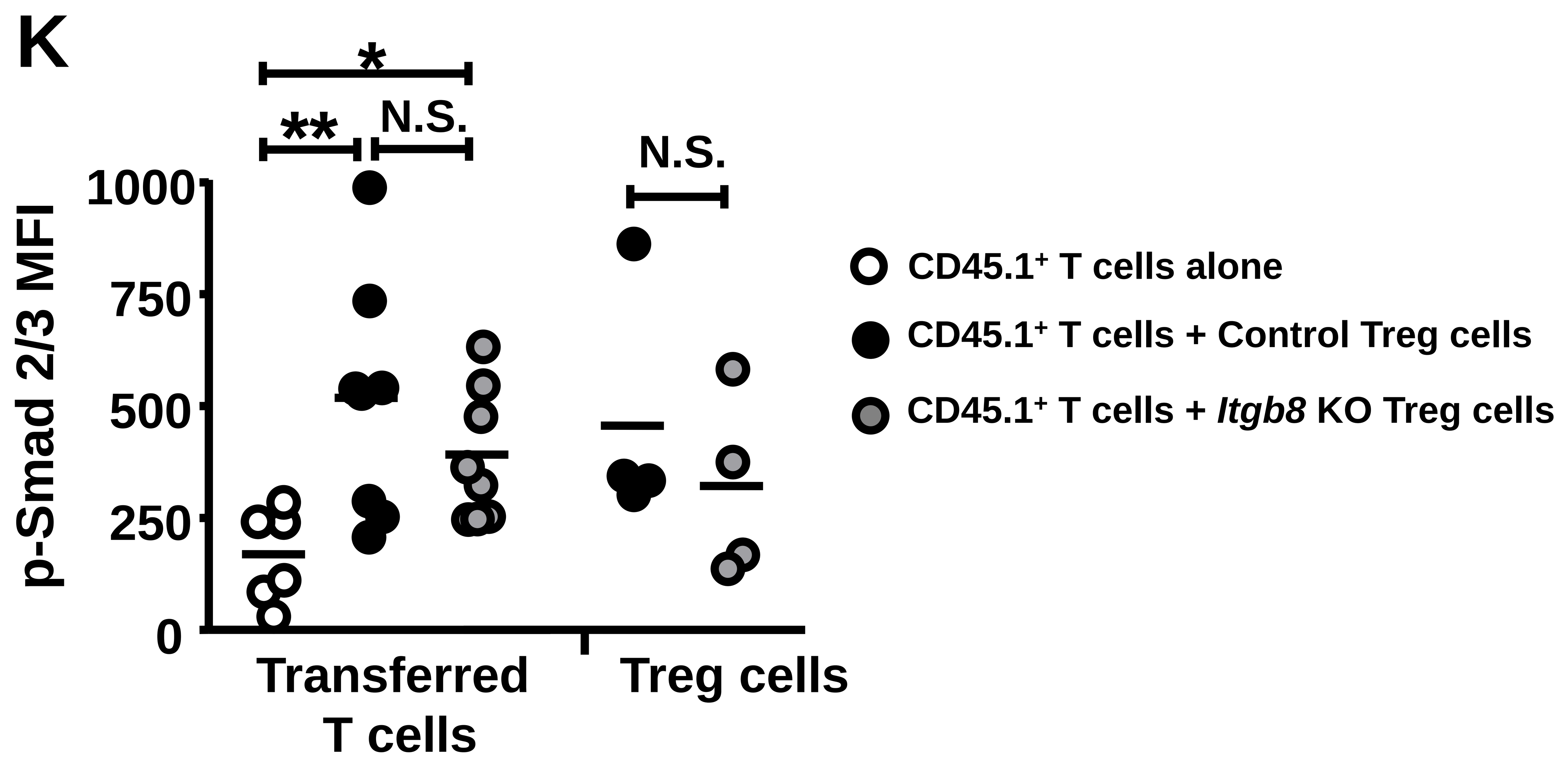

FIGURE S5 (related to Figure 7)

A

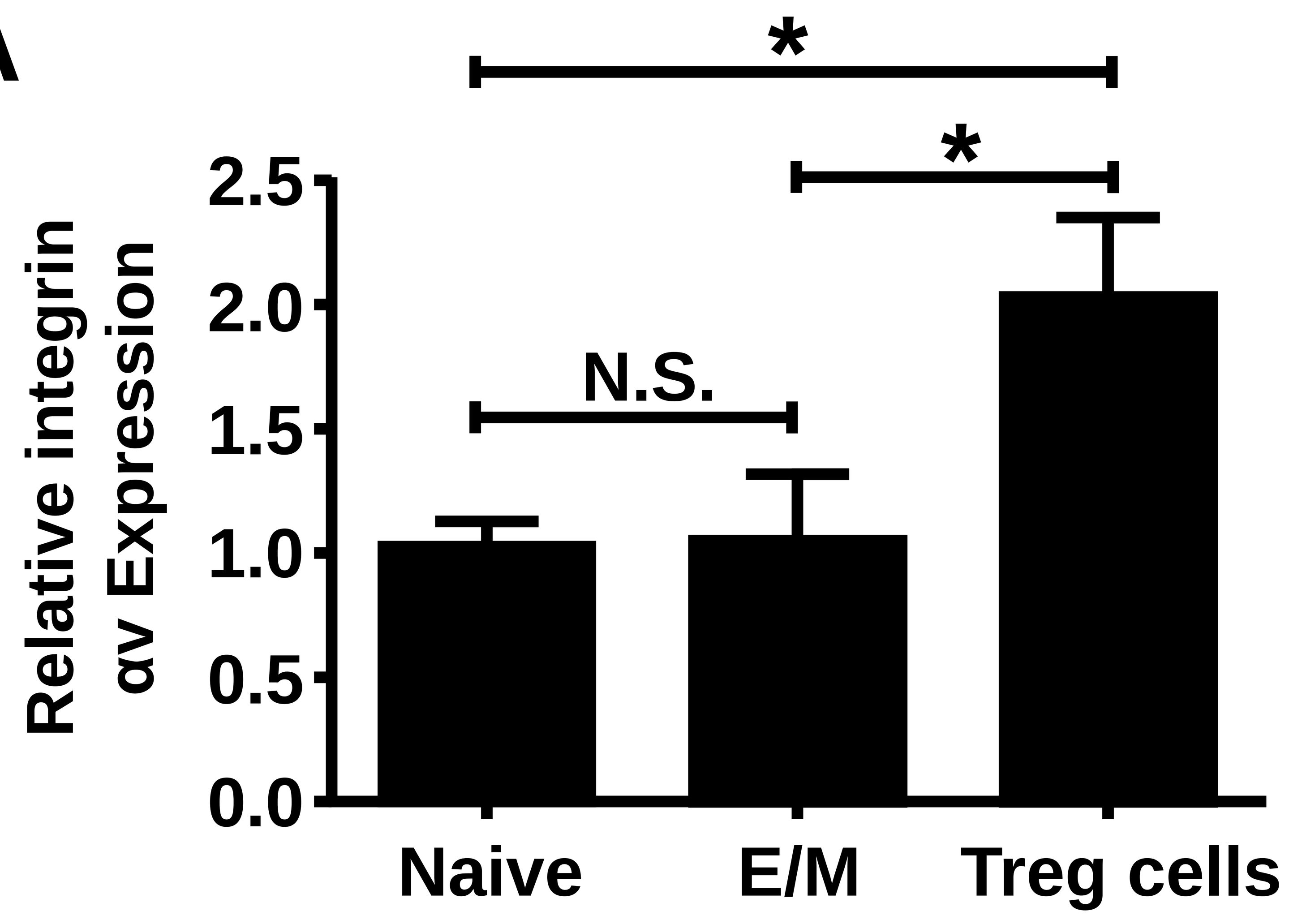

B

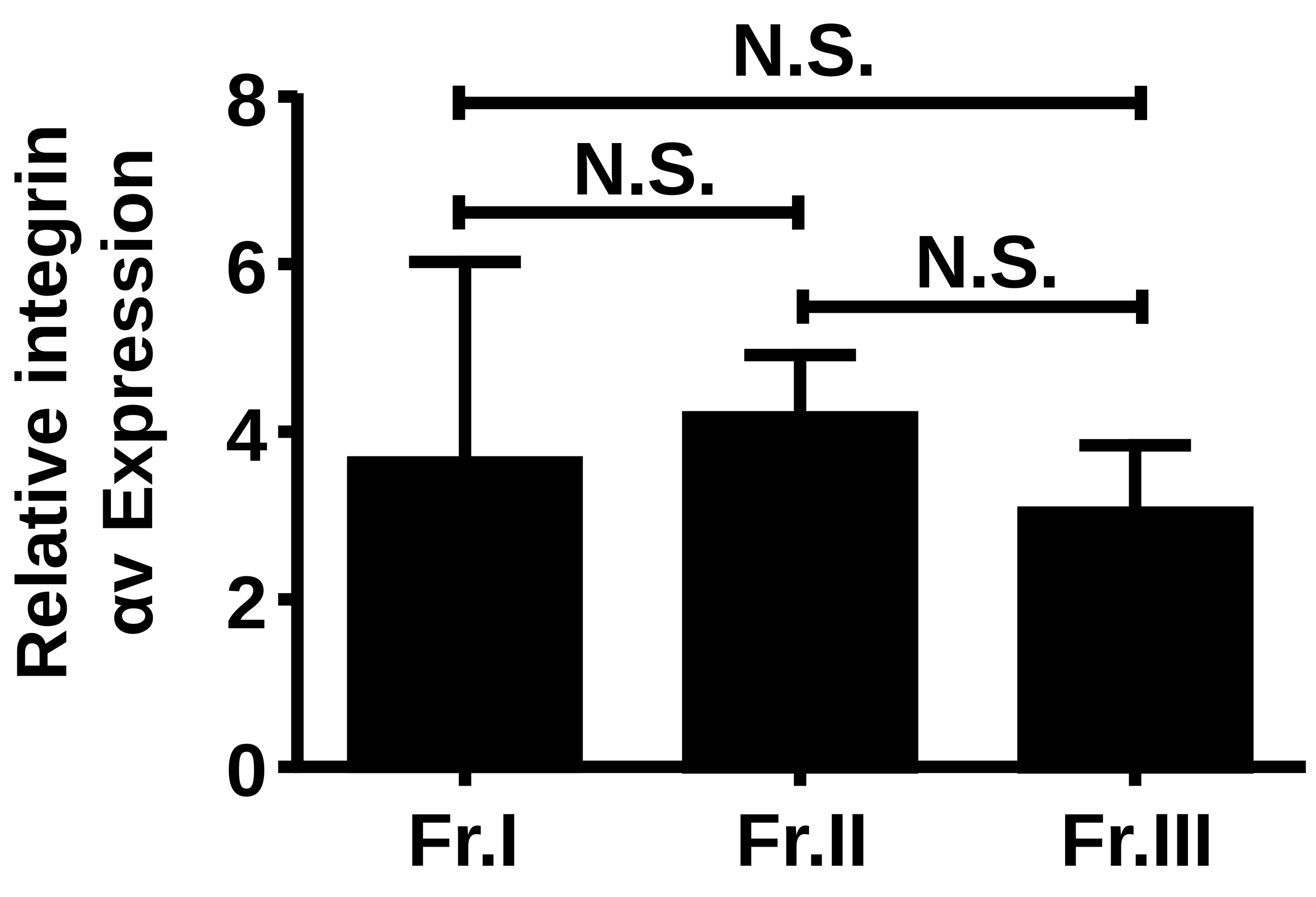

Table S1 (related to Figure 6)

	Control (YFP-Cre$^-$) mean/*MFI* (±SEM)			*Itgb8* KO (YFP-Cre$^+$) mean/*MFI* (±SEM)			Control (Foxp3$^+$, YFP-Cre$^+$) mean/*MFI* (±SEM)			Control (YFP-Cre$^-$) vs. *Itgb8* KO (YFP-Cre$^+$) p value			Control (YFP-Cre$^+$) vs. *Itgb8* KO (YFP-Cre$^+$) p value		
	Spleen	mLN	LILP	Spleen	mLN	LILP	Spleen	mLN	LILP	spleen	mLN	LILP	spleen	mLN	LILP
GITR	94.97 (±1.66)	95.45 (±1.34)	85.45(±5.09)	94.76 (±1.50)	95.44 (±1.32)	85.8(±3.99)	90.71 (±4.08)	94.57 (±2.09)	96.06 (±0.51)	0.93	0.99	0.96	0.28	0.73	0.12
LAP	*487*(±62)	*382* (±63)	1007 (±268)	*488* (±68)	*379* (±61)	*812* (±180)	*580* (±56)	*328* (±115)	*437* (±125)	0.99	0.98	0.56	0.41	0.68	0.22
GARP	*292* (±25)	*167* (±28)	*297* (±83)	*372* (±30)	*170* (±25)	*273* (±71)	*360* (±22)	*126* (±32)	*146* (±42)	0.38	0.92	0.84	0.50	0.33	0.29
CD25	61.46 (±6.43)	67.41 (±4.31)	75.75 (±5.86)	56.15 (±4.54)	61.44 (±4.04)	77.8 (±3.89)	61.59 (±6.32)	68.14 (±3.11)	75.47 (±9.54)	0.52	0.34	0.78	0.51	0.32	0.79
Lag3	*248* (±18)	*133* (±25)	*293* (±97)	*295* (±22)	*149* (±26)	*303* (±80)	*339* (±24)	*111* (±38)	*307* (±154)	0.13	0.67	0.94	0.23	0.43	0.98
Helios	43.02 (±6.84)	44.19 (±2.83)	36.08 (±1.89)	40.77 (±7.18)	41.17 (±1.03)	30.38 (±2.61)	40.08 (±13.38)	40.93 (±2.25)	33.77 (±9.31)	0.83	0.34	0.11	0.96	0.91	0.65
CTLA-4	37.28 (±4.66)	46.33(±5.65)	81.93(±3.15)	34.87 (±3.25)	44.67 (±5.32)	81.34 (±3.80)	36.6 (±2.89)	56.25 (±6.15)	83.89 (±9.70)	0.68	0.84	0.91	0.74	0.23	0.77
Neuropilin	55.7 (±5.31)	47.75 (±4.84)	57.42 (±6.92)	50.28 (±4.95)	49 (±4.42)	61.51 (±6.97)	64.84 (±4.73)	54.26 (±2.40)	53.68 (±3.30)	0.47	0.85	0.69	0.11	0.46	0.48
ST2	6.33 (±1.11)	3.42 (±0.89)	39.11 (±5.91)	7.24 (±2.55)	4.59(±1.05)	35.58 (±1.50)	8.01 (±1.21)	9.73 (±3.01)	44.22 (±11.73)	0.75	0.42	0.58	0.85	0.08	0.32
T-bet	4.64 (±1.38)	4.36 (±1.41)	20.36 (±4.99)	6.62 (±1.08)	4.91 (±1.46)	15.42 (±2.33)	4.33 (±0.22)	3.28 (±1.11)	18.32 (±3.01)	0.23	0.79	0.39	0.19	0.49	0.49
GATA-3	5.52 (±2.40)	8.65 (1.66)	31.75 (±3.16)	5.50 (±2.19)	8.33 (±2.01)	26.24 (±2.70)	1.58 (±0.26)	8.59 (±3.93)	30.14 (±6.95)	0.99	0.92	0.32	0.27	0.96	0.60
p-Smad 2/3	*245* (±13)	*204* (±11)	*214* (±33)	*247* (±16)	*200* (±4)	*292* (±63)	*280* (±4)	*194* (±4)	*351* (±75)	0.91	0.76	0.30	0.2	0.37	0.59
IFNγ	2.51(±0.52)	1.67 (±0.21)	5.96 (±1.92)	1.83 (±0.30)	2.33 (±0.60)	4.50 (±1.49)	1.94 (±0.43)	2.89 (±1.22)	02.22 (±0.56)	0.28	0.32	0.56	0.84	0.65	0.34
IL-17	0.36 (±0.07)	0.75 (±0.34)	0.37 (±0.16)	0.48 (±0.16)	1.1 (±0.34)	0.52 (±0.10)	0.22(±0.07)	1.19 (±0.68)	0.82 (±0.26)	0.53	0.48	0.43	0.32	0.90	0.22
IL-10	12.68 (±3.71)	10.29 (±2.60)	19.42 (±3.12)	13.04 (±3.36)	8.56 (±1.42)	19.23 (±5.37)	7.46 (±2.13)	7.3 (±1.77)	13.1 (±3.10)	0.94	0.57	0.98	0.31	0.61	0.48

Table S2 (related to Figure 6)

	Control (YFP-Cre^{-}) mean/*MFI* (±SEM)			*Itgb8* KO (YFP-Cre^{+}) mean/*MFI* (±SEM)			Control (Foxp3^{+}, YFP-Cre^{+}) mean/*MFI* (±SEM)			Control (YFP-Cre^{-}) vs. *Itgb8* KO (YFP-Cre^{+}) p values			Control (YFP-Cre^{+}) vs. *Itgb8* KO (YFP-Cre^{+}) p values		
	Spleen	mLN	LILP	Spleen	mLN	LILP	Spleen	mLN	LILP	spleen	mLN	LILP	spleen	mLN	LILP
**GITR**	**96.59** (±1.30)	**56.30** (±13.98)	**92.75**(±3.02)	**93.99** (±3.50)	**69.10** (±14.56)	**87.17**(±2.95)	**77.37** (±22.02)	**67.97** (±25.71)	**94.69**(±0.57)	**0.50**	**0.54**	**0.22**	**0.31**	**0.97**	**0.13**
**LAP**	***623*** *(±45)*	***848*** *(±132)*	***731*** *(±111)*	***682*** *(±31)*	***1097*** *(±134)*	***775*** *(±102)*	***590*** *(±101)*	***865*** *(±53)*	***976*** *(±51)*	**0.40**	**0.22**	**0.78**	**0.33**	**0.29**	**0.23**
**GARP**	***206*** *(±21)*	***293*** *(±33)*	***227*** *(±12)*	***220*** *(±23)*	***368*** *(±40)*	***239*** *(±37)*	***302*** *(±139)*	***322*** *(±48)*	***213*** *(±11)*	**0.69**	**0.18**	**0.77**	**0.47**	**0.50**	**0.65**
**CD25**	**55.86** (±11.36)	**72.47** (±7.96)	**57.24** (±8.08)	**52.41** (±10.71)	**71.78** (±6.97)	**49.68** (±8.11)	**47.68** (±4.78)	**69.16** (±0.84)	**37.01** (±8.74)	**0.83**	**0.95**	**0.52**	**0.78**	**0.81**	**0.37**
**Lag3**	***134*** *(±15)*	***231*** *(±21)*	***146*** *(±20)*	***161*** *(±28)*	***273*** *(±22)*	***152*** *(±26)*	***205*** *(±95)*	***278*** *(±36)*	***139*** *(±11)*	**0.41**	**0.19**	**0.86**	**0.57**	**0.89**	**0.75**
**Helios**	**88.00** (±2.46)	**73.37** (±6.86)	**67.28** (±5.31)	**86.63** (±2.51)	**79.56** (±4.93)	**63.26** (±3.69)	**76.23** (±9.13)	**62.08** (±6.14)	**65.91** (±1.02)	**0.73**	**0.48**	**0.55**	**0.22**	**0.07**	**0.64**
**CTLA-4**	**50.51** (±8.12)	**53.02** (±7.44)	**87.53** (±2.91)	**36.19** (±8.23)	**52.14** (±7.28)	**81.51** (±3.96)	**26.81** (±6.23)	**55.21** (±8.31)	**75.91** (±4.63)	**0.24**	**0.93**	**0.25**	**0.49**	**0.80**	**0.42**
**Neuropilin**	**58.40** (±4.73)	**66.92** (±9.94)	**66.63** (±3.26)	**60.67** (±4.58)	**75.76** (±7.32)	**63.95** (±3.82)	**59.44** (±3.61)	**82.27** (±0.42)	**65.32** (±3.5)	**0.74**	**0.49**	**0.61**	**0.87**	**0.67**	**0.83**
**ST2**	**16.74** (±6.45)	**18.58** (±4.41)	**22.59** (±2.88)	**21.32** (±8.74)	**12.40** (±1.92)	**21.22** (±4.90)	**28.15** (±20.25)	**20.71** (±14.67)	**25.38** (±8.77)	**0.68**	**0.23**	**0.81**	**0.72**	**0.43**	**0.66**
**T-bet**	**2.28** (±0.90)	**10.32** (±4.52)	**9.24** (±3.25)	**1.50** (±0.52)	**18.71** (±5.2)	**9.12** (±2.25)	**2.2** (±0.45)	**11.05** (±5.68)	**13.23** (±5.87)	**0.46**	**0.25**	**0.98**	**0.41**	**0.40**	**0.44**
**GATA-3**	**3.25** (±1.06)	**27.13** (8.65)	**32.55** (±1.66)	**4.66** (±1.47)	**26.6** (±2.75)	**31.58** (±1.40)	**8.55** (±3.43)	**33.90** (±6.62)	**38.14** (±14.32)	**0.66**	**0.96**	**0.80**	**0.43**	**0.44**	**0.59**
**p-Smad 2/3**	***534*** *(±34)*	***597*** *(±62)*	***421*** *(±118)*	***513*** *(±28)*	***612*** *(±58)*	***362*** *(±88)*	***392*** *(±110)*	***712*** *(±73)*	***285*** *(±74)*	**0.64**	**0.87**	**0.69**	**0.19**	**0.34**	**0.50**
**IFNγ**	0.79 (±0.18)	**1.27** (±0.70)	**0.05** (±0.03)	**1.05** (±0.38)	**8.96** (±5.60)	**0.09** (±0.04)	**1.74** (±0.97)	**2.50** (±0.61)	**0.2** (±0.05)	**0.56**	**0.20**	**0.51**	**0.44**	**0.46**	**0.20**
**IL-17**	**0.41** (±0.18)	**1.72** (±1.56)	**02** (±0.15)	**0.45** (±0.38)	**0.20** (±0.06)	**0.04** (±0.03)	**0.11** (±0.05)	**0.36** (±0.09)	**0.09** (±0.03)	**0.93**	**0.35**	**0.32**	**0.57**	**0.17**	**0.33**
**IL-10**	**11.58** (±2.87)	**9.13** (±4.56)	**21.10** (±4.16)	**6.48** (±2.84)	**6.75** (±4.99)	**28.57** (±9.25)	**2.07** (±0.85)	**1.31** (±10.26)	**25.07** (±9.25)	**0.24**	**0.50**	**0.44**	**0.33**	**0.39**	**0.80**
